# Drought stress tolerance strategies revealed by RNA-Seq in two sorghum genotypes with contrasting WUE

**DOI:** 10.1186/s12870-016-0800-x

**Published:** 2016-05-21

**Authors:** Alessandra Fracasso, Luisa M. Trindade, Stefano Amaducci

**Affiliations:** Department of Sustainable Crop Production, Università Cattolica del Sacro Cuore, Via Emilia Parmense, 84, 29122 Piacenza, Italy; Wageningen UR Plant Breeding, Wageningen University and Research Centre, 6708 PD Wageningen, The Netherlands

**Keywords:** RNA-Seq, Drought stress, *Sorghum bicolor*, Water Use Efficiency, Drought tolerance

## Abstract

**Background:**

Drought stress is the major environmental stress that affects plant growth and productivity. It triggers a wide range of responses detectable at molecular, biochemical and physiological levels. At the molecular level the response to drought stress results in the differential expression of several metabolic pathways. For this reason, exploring the subtle differences in gene expression of drought sensitive and drought tolerant genotypes enables the identification of drought-related genes that could be used for selection of drought tolerance traits. Genome-wide RNA-Seq technology was used to compare the drought response of two sorghum genotypes characterized by contrasting water use efficiency.

**Results:**

The physiological measurements carried out confirmed the drought sensitivity of IS20351 and the drought tolerance of IS22330 genotypes, as previously studied. The expression of drought-related genes was more abundant in the drought sensitive genotype IS20351 compared to the tolerant genotype IS22330. Under drought stress Gene Ontology enrichment highlighted a massive increase in transcript abundance in the sensitive genotype IS20351 in “response to stress” and “abiotic stimulus”, as well as for “oxidation-reduction reaction”. “Antioxidant” and “secondary metabolism”, “photosynthesis and carbon fixation process”, “lipids” and “carbon metabolism” were the pathways most affected by drought in the sensitive genotype IS20351. In addition, genotype IS20351 showed a lower constitutive expression level of “secondary metabolic process” (GO:0019748) and “glutathione transferase activity” (GO:000004364) under well-watered conditions.

**Conclusions:**

RNA-Seq analysis proved to be a very useful tool to explore differences between sensitive and tolerant sorghum genotypes. Transcriptomics analysis results supported all the physiological measurements and were essential to clarify the tolerance of the two genotypes studied. The connection between differential gene expression and physiological response to drought unequivocally revealed the drought tolerance of genotype IS22330 and the strategy adopted to cope with drought stress.

**Electronic supplementary material:**

The online version of this article (doi:10.1186/s12870-016-0800-x) contains supplementary material, which is available to authorized users.

## Background

Drought is the most important abiotic stress in terms of limiting crop productivity worldwide. Water availability is, therefore, of primary importance for a non-limiting crop production in the current changing global climate scenario. The slogan “more crop per drop” [1] was the track for crop improvement in water limited environments aiming to address the growing demand for water, food and commodities (such as energy) of the growing world population [2].

Among the C4 cereals, *Sorghum bicolor* is the species most suited to environments that are prone to drought. Its tolerance to drought is a consequence of morphological and anatomical characteristics (thick leaf wax, deep root system) and physiological responses (osmotic adjustment, stay green, quiescence) [3]. The high genetic variability among sorghum genotypes and the relatively small size of its genome make this cereal a good model for the identification of drought related genomic regions and genes valuable to unravel the high complexity of drought tolerance related traits [4, 5]. Several sorghum linkage maps, including high density maps [6], have been built using different types of DNA markers [7, 8]. Different genomic regions related to drought tolerance at pre-flowering and post-flowering stage were identified [9] but it was the availability of the sorghum genome sequence [4] that has enabled the monitoring of the genome-wide gene expression profile at a single time in response to several abiotic stresses through microarray or RNA-Seq analysis [3, 10–12]. These studies resulted in the identification of drought stress responsive genes and their regulatory elements.

Several transcriptomics studies were carried out on sorghum using RNA-Seq analysis to monitor gene expression in response to osmotic stress and abscisic acid [3], to provide a *S. bicolor* expression atlas on the dynamic genotype-specific expression profiles [13], or to identify genome-wide SNPs that can potentially enhance genetic analysis and the application of molecular markers in sorghum genomics and breeding [14]. In addition to physiologic or agronomic approaches, genomics offer new opportunities for dissecting quantitative traits into their single determinants (quantitative trait loci, QTLs) paving the way to marker-assisted selection (MAS) or direct gene editing via genetic engineering [15].

Drought stress elicits a wide range of responses in plants [16]. It increases oxidative damage in chloroplasts [17, 18], reduces photosynthesis [19–21], limits metabolic reactions [22], triggers sugar catabolism, in order to provide osmotically active compound and signal molecules [23–25], and modifies cellular lipid composition [26]. To cope with drought stress, plants have developed various strategies, such as generation of larger and deeper root systems [27], regulation of stomatal closure to reduce water loss [28], accumulation of compatible solutes and protective proteins [29], and an increase in the level of antioxidants [30]. Identification of drought resistant traits was frequently labelled as “complex” although we already know the results of all the modifications adopted by plants to cope with drought stress [31].

In this study we have furthered extended the knowledge on the drought response of two sorghum genotypes through transcriptomic analysis [32]. A massive parallel sequencing of RNA (RNA-Seq) on the Illumina platform was used to provide a thorough scenario on the whole sorghum transcriptome in response to drought stress. Several categories of key genes involved in drought response have been identified.

## Results

### Physiological responses to drought stress

**Fig. 1:**
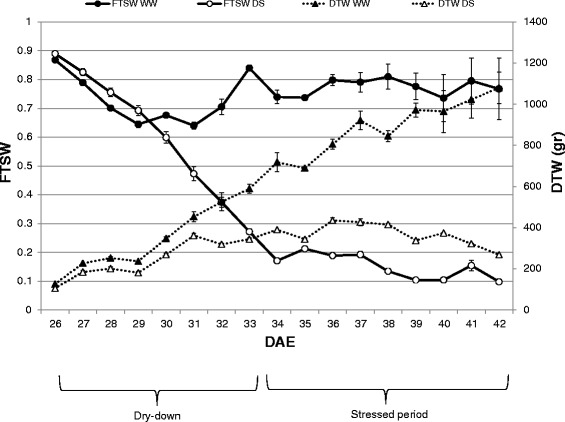
Trend of FTSW and daily transpired water during the dry-down experiment. On the left axis with circles symbols the trend of FTSW during the dry-down: with full circles the WW plants and with the empty circles the DS ones. On the right axis with triangles the daily transpired water: full triangles for the WW plants and empty triangles for the DS ones. DAE = days after emergence. Mean of 10 plants ± SE

Twenty sorghum plants (ten per each genotype) were subjected to severe drought stress by withholding water from 26 DAE (Days After Emergence) until 34 DAE when 0.2 FTSW (Fraction of Transpirable Soil Water) was reached in all the stressed plants (Fig. 1, solid line, white dots). Subsequently the stressed plants were kept at 0.2 FTSW by irrigating daily for nine days, while the control plants were kept at FTSW values higher than 0.6 for the entire duration of the experiment (Fig. 1, solid line, full dots). The daily transpired water (DTW) was under 400 gr for the stressed plant, while it was up to 1000 gr for the control plants (Fig. 1, dotted lines).

Leaf area, chlorophyll fluorescence parameters (maximum quantum yield, Fv/Fm, the photosystem II efficiency, ΦPSII, and non-photochemical quenching, qNP) and gas exchange measurements (photosynthetic rate, Pn, and transpiration E) were quantified for the entire duration of the experiment (data not shown).

The decreased FTSW led to a reduction in RWC (Relative Water Content) values and these changes were greater in the sensitive genotype IS20351 than in the tolerant genotype IS22330 (Table 1). Drought stress also dramatically reduced chlorophyll fluorescence and photosynthetic rate. Under stress conditions the tolerant genotype IS22330 showed a significantly higher value of Fv/Fm than the sensitive genotype IS20351 (Table 1). The same trend was observed for ΦPSII: 0.36 and 0.28 for the tolerant and the sensitive genotype, respectively. In contrast, the qNP under drought stress was higher in the sensitive genotype IS20351 than in the tolerant genotype IS22330 (Table 1).

**Table 1:** Physiological responses of sorghum genotypes to drought stress

Genotype	Condition	FTSW	RWC	Chlorophyll fluorescence			Gas exchange			
				Fv/Fm	ΦPSII	qNP	Pn	E	WUE_i_	WUE_a_
		%	%				μmol m^−2^ s^−1^	mmol m^−2^ s^−1^	μmol mol^−1^	g/l
IS20351	WW	0.70	92.7	0.803^a^	0.50^a^	0.18^a^	31.2^a^	4.58^b^	6.38^a^	3.31^b^
	DS	0.2	78.4	0.779^c^	0.28^c^	0.15^b^	19.8^c^	5.56^a^	3.56^c^	3.26^b^
IS22330	WW	0.80	92.9	0.804^a^	0.52^a^	0.11^b^	30.4^a^	4.51^b^	6.74^a^	3.74^ab^
	DS	0.2	88.4	0.791^b^	0.36^b^	0.08^c^	24.1^b^	4.93^b^	4.88^b^	4.23^a^
LSD _(0.05)_			7.76	0.006	0.08	0.002	2.4	0.58	0.28	0.59
P		<0.0001	<0.01	<0.0001	<0.05	<0.0001	<0.0001	<0.001	<0.01	<0.05

Analysis of relative water content (RWC), chlorophyll fluorescence (Fv/Fm, FPSII and qNP), gas exchange (Photosynthetic rate, Pn, and Transpiration, E), intrinsic (WUEi) and agronomic WUE (WUEa) in sorghum plants in well-watered (WW) and drought stress (DS) conditions at vegetative stage of 9th leaf. Values followed by the same letter are not statistically significant at LSD test p < 0.05 performed on the interaction genotype× irrigation.

**Fig. 2:**
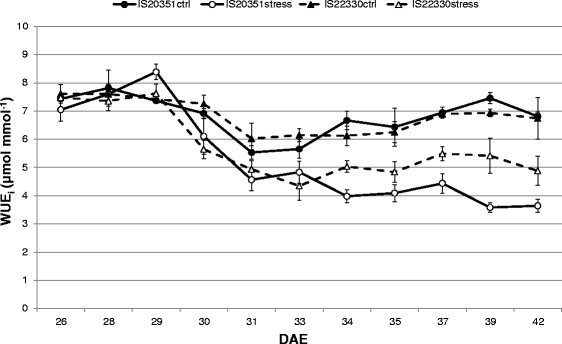
Trend of WUE_i_ calculated during the dry down experiment. Circles represent the sensitive genotype IS20351 and triangles the tolerant IS22330. For both the genotypes the full symbols represents the WW plants whilst the empty symbols represent the DS ones. Mean of 10 plants ± SE

Drought stress affected Pn in both the genotypes differently; the sensitive genotype IS20351 had a greater reduction in Pn (36.5 %) while the tolerant genotype IS22330 showed a Pn reduction of 20.7 %. Transpiration (E) did not differ between the WW (Well-Watered) and DS (Drought-Stressed) plants of the tolerant genotype IS22330, while there was a statistically significant difference between the WW and DS plants of the sensitive genotype IS20351. The intrinsic water use efficiency (WUE_i_) decreased linearly for the DS plants of both genotypes from the beginning of the experiment (26 DAE) until harvest (42 DAE), while the WW plants kept their WUE_i_ close to 6 μmol mmol^−1^ (Fig. 2). WUE_i_ of DS plants of the tolerant genotype IS22330 was significantly higher than that of DS plants belonging to the sensitive genotype IS20351 during the stress period (*p* < 0.05) (Fig. 2). The agronomic water use efficiency (WUE_a_), calculated at harvest, was higher for the tolerant genotype IS22330 (4.23 g/l) than for the sensitive genotype IS20351 (3.26 g/l), thereby confirming the trend highlighted by WUE_i_.

### Drought stress reveals different intergenic transcripts and novel splice sites

Transcription profiles of IS20351 and IS22330 under well-watered (WW) and drought-stressed (DS) conditions were explored using the Illumina Genome Analyzer deep sequencing. Three biological replicates were analysed for each condition, resulting in twelve samples. In total, 0.56 billion clean reads, each 100 nucleotides long, were generated, with approximately 47 million clean reads from each sample. The reads mapping to the reference genome were categorised into two classes: uniquely mapped reads, that are reads that map to only one position in the reference genome, and multi-position match, that are reads mapping to more than one position in the reference genome (Table 2). The assembled transcripts were mapped on the genome: on average 72 % were known transcripts, 10 % were novel transcripts and 18 % were intergenic transcripts (Table 3).

**Table 2:** Number of reads sequenced and mapped with SOAPaligner/SOAP2

Genotype	Treatment	Total Reads	Total Unmapped Reads	Total Mapped Reads	Unique match	Multi-position match
IS20351	WW	47090292	11993194	35097098	32702251	2394847
	DS	46866452	11969692	34896760	32188700	2708060
IS22330	WW	47504944	11856843	35648101	32660622	2987479
	DS	46840269	11086330	35753938	32549143	3204795

The numbers of unique mapped reads plus the multi-position match equals the total number of mapped reads in well-watered (WW) and drought stress (DS) conditions

**Table 3:** Classification of transcript produced in sorghum leaves under well-watered (WW) and drought stress (DS) conditions

Genotype	Treatment	Total Mapped Reads	Match to known transcripts	Intergenic transcripts	Novel Transcripts	Alternative Splicing Events
IS20351	WW	75 %	73 %	18 %	9 %	24178
	DS	74 %	71 %	20 %	9 %	24367
IS22330	WW	75 %	72 %	18 %	10 %	20498
	DS	76 %	72 %	18 %	10 %	24304

Percentage of total mapped reads on the reference genome, percentage of match with known transcripts, with intergenic transcripts and novel transcripts identified, and alternative splicing events identified

Drought stress induced alternative splicing events (ASE) in the two genotypes (Table 3): in the sensitive genotype IS20351 no difference in ASE were found, while in the tolerant genotype IS22330 the ASE were increased by 18 %.

### Drought stress triggers differential expression of particular genes and GO classes

**Fig. 3:**
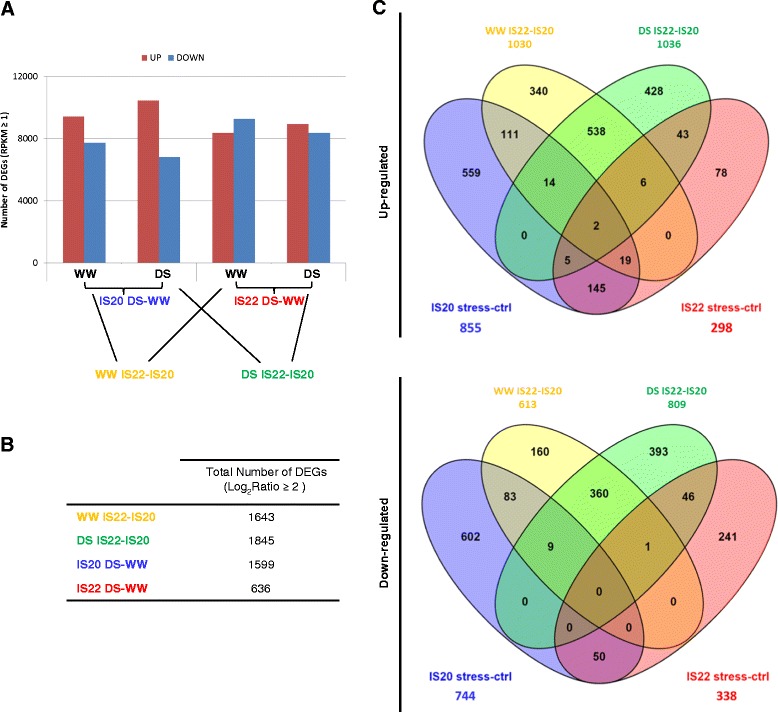
Comparison under study. **a** Number of DEGs (RPKM) in each pairwise comparison. Blue and red bar are up- an down-regulated genes respectively expressed in well-watered (WW) and drought stressed (DS) conditions in the genotypes IS20351 (IS20) and IS22330 (IS22). **b** Total number of DEGs that passed the cut-off of Log_2_ FC >2 in each comparison. In yellow the number of DEGs resulting from the comparison between IS20351 and IS22330 in well-watered (WW) conditions, in green the number of DEGs resulting from the comparison between the two genotypes under drought stress (DS) conditions; in blue the numbers of DEGs in response to drought stress in IS20351 and in red the number of DEGs in response to drought stress in IS22330. **c** Venn diagram showing the numbers of up- and down- regulated genes resulted from the four comparison performed. The number of up- or down- regulated genes shared among the four comparison is represented by overlapping circles

Each condition was represented by three biological replicates, resulting in eighteen pairwise comparisons between control and stressed plants of the two genotypes. The transcript abundance of each gene was calculated as reads per kilobase transcriptome per million mapped reads (RPKM) (Fig. 3a). This value was used to determine the differential expression analysis as Log_2_ ratio between DS and WW plants per genotype and between the two genotypes under WW and DS conditions. Four comparisons were analysed in this study: i) the genotypes IS20351 and IS22330 under WW conditions (WW IS22-IS20 in yellow), ii) the genotypes IS20351 and IS22330 under DS conditions (DS IS22-IS20 in green), iii) the genotype IS20351 in response to DS conditions (IS20 DS-WW in blue), iv) the genotype IS22330 in response to DS conditions (IS22 DS-WW in red).

After applying a stringent cut-off (see Methods section), the comparison of genotypes IS20351 and IS22330 under WW conditions identified 1643 differentially expressed genes (DEGs), and the comparison of genotypes IS20351 and IS22330 under DS conditions identified 1845 DEGs. 1599 genes were differentially expressed in IS20351 in response to drought stress, whilst only 636 were differentially expressed in IS22330 (Fig. 3b). Venn diagrams highlight the overlap of DEGs between each pairwise comparison (Fig. 3c).

Comparison between IS22330 and IS20351 under WW conditions (Fig. 3c in yellow) resulted in 1030 up-regulated genes and 613 down-regulated genes. Only 340 genes were uniquely up- and 160 genes down-regulated in IS22330 in these conditions. The singular enrichment analysis (SEA), carried out with AgriGO software (http://bioinfo.cau.edu.cn/agriGO/index.php) on the 340 up-regulated genes, highlighted 34 GO terms significantly enriched: “aromatic compound biosynthetic process” (GO:0019438), “secondary metabolic process” (GO:0019748), and “flavonoid biosynthetic process” (GO:0009812) in the cellular processes category; “glutathione transferase activity” (GO:0004364), “oxygen binding” (GO:0019825), “UDP-glucosyltransferase activity” (GO:0035251) in molecular functions category (Additional file 1: Table S1). “Apoptosis” (GO:0006915) and “oxidoreductase activity” (GO:0016491) were the most enriched GO terms in the biological processes and molecular function categories among the 160 uniquely down-regulated genes expressed in WW conditions in IS22330 (Additional file 1: Table S2).

The comparison between the two genotypes under DS conditions resulted in 1036 up- and 809 down-regulated genes. Among these genes, only 428 and 393 were uniquely up- and down- regulated in the genotype IS22330 in comparison to IS20351. “Regulation of DNA replication” (GO: 0006275), “cell death” (GO:0008219), “regulation of cell growth by extracellular stimulus” (GO:0001560), “secondary metabolic processes” (GO:0019748) including “terpenoids biosynthetic process” (GO:0016114), “glutathione transferase activity” (GO:0004364) and “pre-replicative complex” (GO:0005656) (Additional file 1: Table S3) were the most enriched GO terms among the 75 identified after SEA of the 428 up-regulated genes. Among the 393 down-regulated genes 24 GO terms were significantly enriched: “lipid localization” (GO:0010876), “apoptosis” (GO:0006915), “flavonol biosynthetic process” (GO:0051555), “electron carrier activity” (GO:0009055) and “heme binding” (GO:0020037) (Additional file 1: Table S4).

**Fig. 4:**
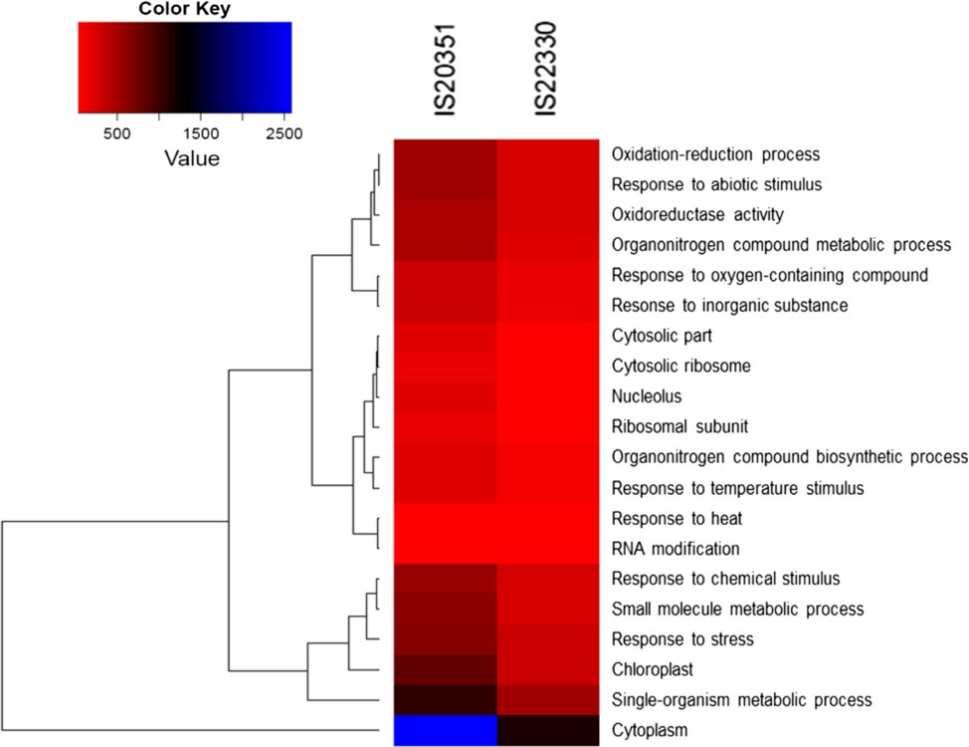
Heat map showing the 20 common GO terms enriched under drought stress in sorghum leaves of IS20351 and IS22330. The cluster frequency was used as a parameter for the parametric analysis of gene enrichment analysis. The figure was generated using R software, Limma package

The main difference between the two genotypes was in the total number of genes differentially expressed in response to drought stress: 1599 for the sensitive IS20351 and 636 for the tolerant IS22330. The SEA analysis, performed on all the 1599 and 636 DEGs expressed in response to drought in the genotypes IS20351 and IS22330, showed 197 significantly enriched GO terms (*p*-value <0.05) in the sensitive genotype IS20351 while 34 in the tolerant IS22330. Twenty GO terms were enriched in both the genotypes in response to drought stress and are represented in the heat map (Fig. 4). “Response to heat”, “RNA modification”, “cytosolic part” and “ribosomal subunit” GO terms were enriched with the same extent in both the genotypes. Different GO enrichment was recorded between IS203351 and IS22330 for “oxidation-reduction process”, “response to abiotic stimulus”, “oxidoreductase activity”, “response to chemical stimulus”, “small molecule metabolic process”, “response to stress”, “chloroplast”, “single-organism metabolic process” and “cytoplasm component”. All these GO terms were more enriched in IS20351 than in IS22330.

Between the two genotypes there were 145 common up-regulated genes in response to drought stress and 50 common down-regulated genes (Fig. 3c). The SEA performed on these common DEGs highlighted 11 enriched GO terms belonging to biological processes: “response to abscisic acid stimulus” (GO:0009737), “response to water deprivation” (GO:0009414), “photosynthesis, light reaction” (GO:0019684) were the most enriched GO (Additional file 1: Table S5).

The SEA analysis performed with AgriGO on the unique up-regulated genes of IS20351and IS22330 (respectively 559 and 78 genes) highlighted 74 enriched GO terms in IS20351 and and 6 enriched GO terms IS22330. The cross comparison of SEA (http://bioinfo.cau.edu.cn/agriGO/analysis.php?method=compare) highlighted 6 common GO terms (Additional file 1: Table S6). The SEA analysis performed on the unique down-regulated genes (602 and 241 for IS20351 and IS22330 respectively) highlighted 166 and 32 significantly enriched GO terms in IS20351 and IS22330 respectively; after the cross comparison of SEA only 6 resulted as being common to both genotypes (Additional file 1: Table S7).

### Drought stress affects different pathways

**Fig. 5:**
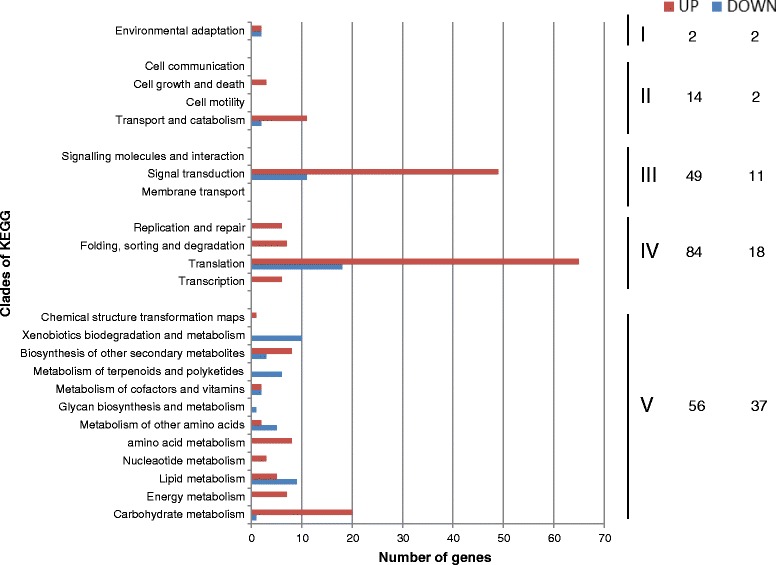
Number of up- and down-regulated genes in each clade of the KEGG pathway maps. The 171 unigenes were assigned 112 KEGG pathways within 24 clades under five major categories: “organismal systems” (I), “cellular processes” (II), “environmental information processing” (III), “genetic information processing” (IV), “metabolism” (V). Per each clades are shown the up- (in red) and the down- (in blue) regulated genes

The KEGG pathway analysis was performed to assign the related biological pathways in which DEGs were involved. One-hundred and seventy-one genes, uniquely expressed in response to drought stress in both the genotypes, were assigned to 112 different KEGG pathways belonging to 24 clades under five major KEGG categories including ‘organismal system’ (I), ‘cellular process’ (II), ‘environmental information processing’ (III), ‘genetic information processing’ (IV), and ‘metabolism’ (V) (Fig. 5). Gene-set enrichment analysis showed that translation, signal transduction and carbon metabolism were the top three up-regulated pathways represented by the genes uniquely expressed in response to drought stress; metabolism pathways (V) and signal transduction were, on the other hand, the most enriched down-regulated pathways (Fig. 5).

**Fig. 6:**
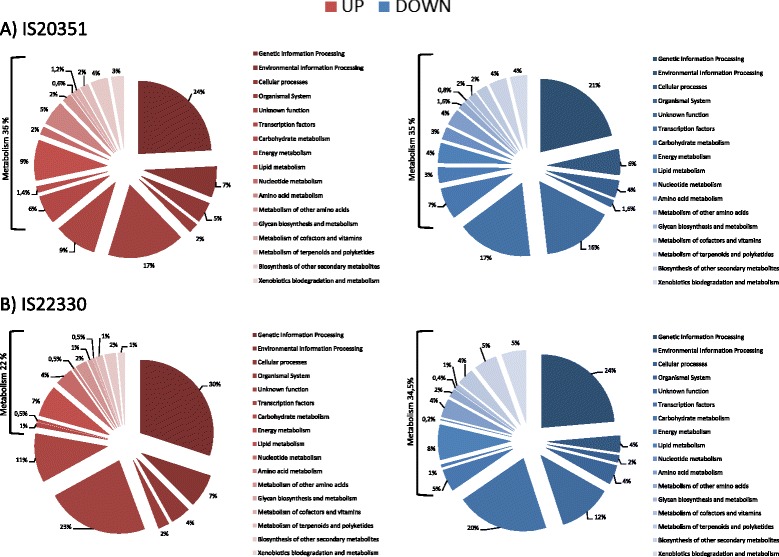
Distribution in KEGG pathways of the unique up- and down-regulated genes in response to drought for the genotype IS20351 and IS22330. Pie charts showing the percentage of genes up- (*in red*) and down- (*in blue*) regulated in response to drought stress for the genotypes IS20351 (**a**) and IS22330 (**b**)

KEGG pathway analysis was also performed on the genes that were uniquely up- and down-regulated in response to drought stress in both genotypes (Fig. 6). Transcription factors, ‘environmental information processing’ pathways, and pathways related to ‘cellular processes’ and ‘organismal system’ remained unchanged among the uniquely up-regulated genes (Fig. 6 in red). The most striking differences in the transcriptomic profiles of the two genotypes in response to drought were mainly in the ‘metabolism’ pathways (that were up-regulated by 36 % in IS20351 and 22 % in IS22330), in the ‘genetic information processing’ pathway (that was up-regulated to a greater extent in IS20351) and in the number of genes not assigned to pathways (Fig. 6 in red). Focusing on the up-regulated ‘metabolism’ pathways, the tolerant genotype IS22330 showed a two-fold (or greater) enrichment in the metabolism of other amino acids, the nucleotide metabolism, the glycan biosynthesis metabolism and the lipid metabolism compared to the sensitive genotypes IS20351 (Fig. 6 in red). Amino acid metabolism, carbohydrate metabolism and energy metabolism were more enriched in the sensitive genotype IS20351 than in the tolerant genotype IS22330 (Fig. 6 in red).

The ‘metabolism’ pathways of IS20351 and IS22330 were down-regulated to the same degree in response to drought stress (Fig. 6 in blue). ‘Cellular processes’ pathways represented 4 % of the down-regulated genes in IS20351 and 2 % in IS22330 (Fig. 6 in blue). ‘Organismal system’ pathways, ‘genetic information processing’ pathways and transcription factors were down-regulated to a greater extent in the tolerant genotype IS22330 (Fig. 6 in blue). Among the down-regulated ‘metabolism’ pathways, energy metabolism, nucleotide, cofactors and vitamins metabolism, glycan biosynthesis and metabolism, and carbohydrate metabolism pathways were down-regulated with a higher frequency in the sensitive genotype IS20351 than in the tolerant IS22330 (Fig. 6 in blue).

### Drought stress response of sorghum transcriptome

**Fig. 7:**
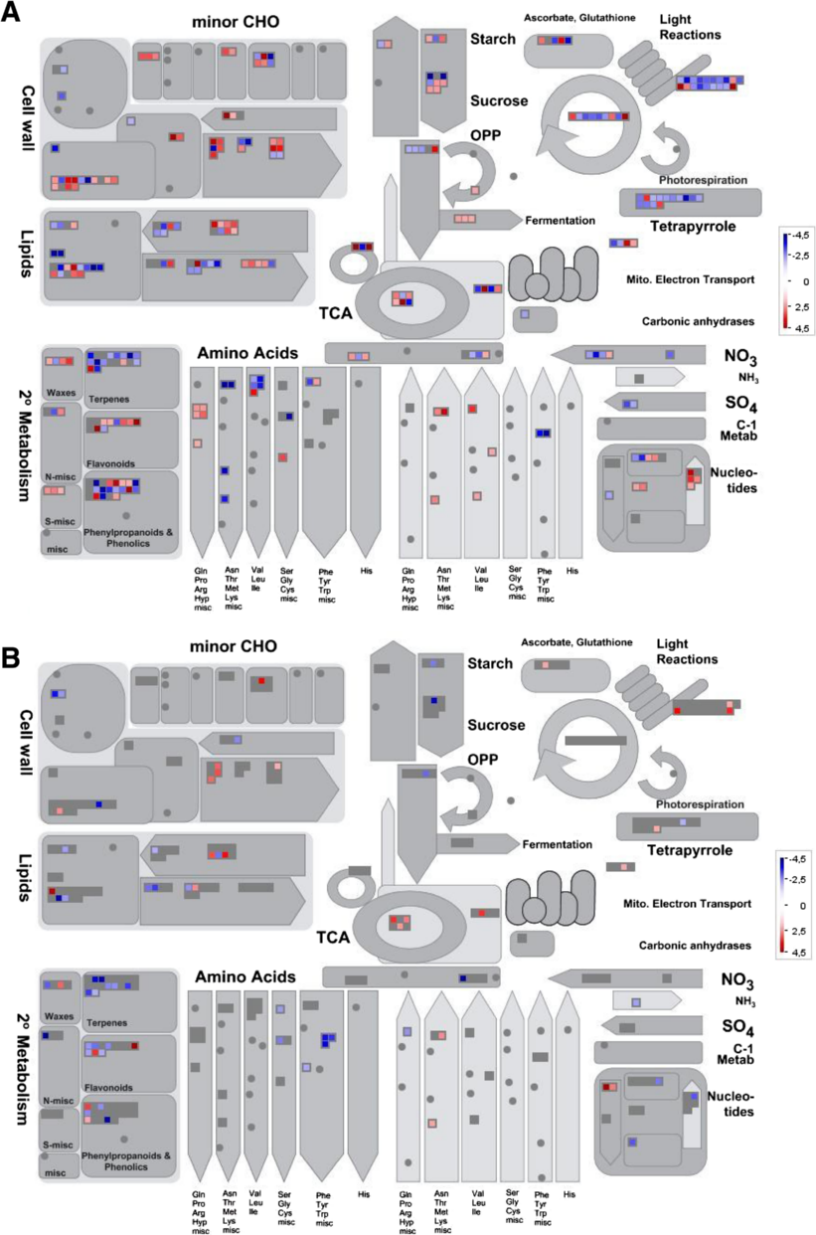
Distribution of up- (*in red*) and down- (*in blue*) regulated genes in metabolic pathways in response to drought stress for IS20351 and IS22330. Drought mediated expression changes in the metabolic pathways in leaves of IS20351 (**a**) and IS22330 (**b**). The figure was generated using MapMan and shows DEGs that passed the cut-off of Log_2_ FC >2

The MapMan software (3.5.1R2) [33] was used to show a pathway overview of 1599 and 636 DEGs expressed in response to drought stress and it was selected for its capacity to show statistically significant drought mediated gene expression data for the sensitive genotype IS20351 (Fig. 7a) and the tolerant genotype IS22330 (Fig. 7b). Three main aspects were selected for a deeper evaluation of drought tolerant traits: the antioxidant and secondary metabolism pathways, light reaction and carbon fixation pathways, lipid and carbon metabolism.

### Response of antioxidant and secondary metabolism related genes

DEGs related to antioxidant and secondary metabolism were analysed together because of the strong relationship between the capacity to scavenge ROS through antioxidant genes and metabolites derived from the secondary metabolism.

Seventeen DEGs were identified in the sensitive genotype IS20351 in response to drought: 5 were up-regulated and 12 down-regulated (Additional file 2: Table S1). In the tolerant genotype IS22330, in the same condition, only 4 DEGs were found and three of them were up-regulated. The sb09g025730.2 gene showed a peculiar behaviour; it was up-regulated in the tolerant genotype IS22330 and dramatically down-regulated in the sensitive IS20351. The sb06g001970.1 gene was up-regulated in the sensitive genotype IS20351 and remained unchanged in the tolerant IS22330. In contrast, the sb09g001690.1 gene was up-regulated in the tolerant IS22330 and its expression remained unchanged in the sensitive IS20351.

Drought affected the secondary metabolism in both sorghum genotypes. Fifty DEGs were found in the sensitive genotype IS20351 and 27 in the tolerant IS22330 (Additional file 2: Table S1). In the sensitive genotype IS20351, about the same number of genes were up- and down-regulated (25), whilst in the tolerant genotype IS22330 the down-regulated genes were more than the up- regulated ones; 20 and 7, respectively (Additional file 2: Table S1). Among the down-regulated genes, the isoprenoids and phenylpropanoids metabolism was the most affected metabolism, with 20 genes in IS20351 and 10 in IS22330. The flavonoids pathway showed a peculiar behaviour being up-regulated by drought in the sensitive genotype IS20351 and down-regulated in the tolerant genotype IS22330. The changes in the secondary metabolism expression pattern, for example the change in the chlorophyll/carotenoids content, was reflected in the fluorescence parameters recorded.

### Response of light reactions and carbon fixation pathways

The photosynthetic pathway was drastically affected by drought in the sensitive genotype IS20351, with 28 genes differentially expressed in response to drought: 19 belong to the light reaction pathway and 9 to the Calvin cycle.

Among the 19 DEGs belonging to the light reaction pathway, 15 genes were down-regulated in response to drought (Additional file 2: Table S1): 8 code for protein belonging to the light harvesting complex I or II (LHCI and LHCII), 6 code for protein related to photosystem I and II (PSI and PSII) and 1 codes for the gamma subunit of the ATP synthase. Two isoforms of PSII polypeptide subunits were strongly up-regulated together with the electron carrier ferrodoxin in the sensitive genotype IS20351 in response to drought (Additional file 2: Table S1). In the tolerant genotype IS22330 the light reaction pathway was also affected, but to a lower extent. Only three genes belonging to the light reaction pathway were up-regulated in response to drought: 2 implicated in PSII and one in photosynthetic electron transport, the ferrodoxin (Additional file 2: Table S1).

9 genes related to the carbon fixation pathway (Calvin cycle) were differentially expressed in the sensitive genotype IS20351 (Additional file 2: Table S1): 6 were down-regulated by drought and 3 were up-regulated (Sb01g037510.1, Sb06g004280.1 and Sb05g027880.1). In the tolerant genotype IS22330 no genes were differentially expressed in response to drought (Additional file 2: Table S1).

### Lipid and carbon metabolism in response to drought stress

In terms of DEGs the lipid metabolism was more greatly affected in the sensitive genotype IS20351 (Additional file 2: Table S1). In this genotype fatty acid synthesis, elongation and lipid degradation via beta-oxidation cycle were all up-regulated (Additional file 2: Table S1). Phospholipid and sphingolipid syntheses were down-regulated in response to drought (Additional file 2: Table S1). In the tolerant genotype IS22330 the steroids biosynthesis and phospholipase D were up-regulated (Additional file 2: Table S1).

Also the carbon metabolism was more greatly affected by drought in the sensitive genotype IS20351 than in the tolerant IS22330. In IS20351 drought highlighted 12 DEGs: 7 genes belonging to the degradation of starch and sucrose were up-regulated, and 5 genes were down-regulated (Additional file 2: Table S1). In the tolerant genotype IS22330 only 2 genes were down-regulated (Additional file 2: Table S1).

## Discussion

In plants exposure to drought triggers a wide range of responses, ranging from molecular expression, biochemical metabolism to ecosystem level, that involve lots of genes and pathways related to diverse mechanisms [16]. In this study we evaluated these mechanisms through RNA-Seq analysis of two sorghum genotypes subjected to the same extent of drought stress. The responses differed greatly between the sensitive IS20351 and the tolerant IS22330 genotypes in terms of the number of genes and pathways involved in drought stress response, but also in terms of the constitutive expression level of several pathways.

### Constitutive drought tolerance trait

The trend of FTSW, together with the value of the daily transpiration rate, confirmed that the DS plants of both genotypes were subjected to the same environmental conditions and to the same extent of drought stress. In addition, transcriptomics analysis provided unequivocal evidence on RNA modifications triggered by drought stress. “Response to heat” (GO:0009408) and “RNA modification” (GO:0009451) GO terms were enriched to the same extent in both genotypes.

Although the drought stress level applied was equal (0.2 FTSW), the two genotypes responded differently; in IS20351 a significantly higher number of differentially expressed genes (DEGs) was observed than in the tolerant genotype IS22330, resulting in a greater enrichment of GO terms related to drought stress response in IS20351 than in IS22330. The up-regulation of genes under WW conditions of “secondary metabolic process” (GO:0019748), and related GO terms, in the genotype IS22330 confirm its intrinsic tolerance, previously only characterized from a physiological point of view [32]. In this genotype, the constitutive upper level of flavonoids and secondary metabolites led to increased drought tolerance traits according to Winkel-Shirley [34]. Furthermore the “glutathione transferase activity” (GO:000004364) was up-regulated in the tolerant genotype IS22330 confirming the role of the glutathione-S transferase family in improving environmental stress resistance in crops [35].

### Drought tolerance strategies

Drought stress results in a massive production of reactive oxygen species (ROS) [17, 18] that cause oxidative stress. The sequence of events that occur in plant tissues in response to oxidative drought-induced stress was well described by Mano et al. [36]. The antioxidant enzymes constitute the “first line of defence” against ROS and oxidative stress generated by different abiotic and biotic injuries [37, 38]. The activity of these enzymes can be enhanced or repressed depending on the species, genotype, stress duration and severity [39–41]. In the “response to abiotic stimulus” (GO:0009628), “oxido-reductase activity” (GO:0016491) and “response to stress” (GO:0009628) gene ontology categories, genes were more greatly down-regulated by drought in the sensitive genotype IS20351 than in the tolerant IS22330, enabling us to speculate that the tolerant IS22330 had a constitutively higher expression of antioxidant genes that is not affected by drought stress. Experimental evidence showed that the antioxidant enzyme activity might be depressed in excess-light conditions, especially when plants are faced with additional stresses such as drought or temperature [42].

To cope with the oxidative stress caused by drought, genes coding for secondary metabolites such as phenylpropanoids, phenolic compounds and flavonoids, are overexpressed [43]. Phenylpropanoids have the greatest potential to reduce ROS, the polyphenols act as antioxidants to protect plants against oxidative stress [44], flavonoids play different molecular functions, including stress protection in plants [34], and also flavanols were found to be oxidated in response to severe drought in tea plants, suggesting their involvement in plant protection [45]. All these compounds are widely synthetized in response to several abiotic stresses, including drought [46–50]. In wheat and willow leaves an increase in flavonoid and phenolic acids content was observed together with an induction of genes involved in the flavonoid biosynthetic pathway in response to various stresses, including drought [51, 52]. With our study, we confirm that under drought stress the up-regulation of these genes in the sensitive genotype IS20351 was higher than in the tolerant genotype IS22330, whilst a constitutively higher expression of these genes in the tolerant IS22330 under control conditions led to a lower synthesis of stress induced compounds. The accumulation of these compounds and the differential expression of the above mentioned genes remains genotype dependent [53].

Only in the last decade was it hypothesized that flavonoids might also play a role as antioxidant in response to severe excess of light complementing the role of antioxidant enzymes [54–57]. Agati et al. [42] found that flavonoid genes were up-regulated in response to drought in the sensitive genotype IS20351 whilst they were mostly down-regulated in the tolerant IS22330. The biosynthesis of “antioxidant” flavonoids, in fact, increases more in stress sensitive species than in stress tolerant ones [42]. The reason for this lies in the fact that stress sensitive species display a less efficient “first line” of defence against ROS in conditions of stress and they are therefore exposed to a more severe oxidative stress [58, 59]. In any case, the relationship between antioxidant enzymes and flavonoids in response to abiotic and biotic stress it is not yet well clarified [42].

Drought stress induces a decrease in the chlorophyll content, a consequential change in the chlorophyll/carotenoid ratio [60] and an increase in the ratio of violaxanthin-cycle pigment. This results in a reduction of light absorption centres, an enhancement of non-photochemical quenching in order to dissipate the excess of light, and a reduction in photosynthetic rate [19–21]. All these stress-induced physiological modifications (qNP and Pn) were observed to a greater extent in the sensitive genotype IS20351. The physiological response is supported by the observation that a high number of genes involved in the terpenoids and carotenoids biosynthesis were down-regulated in IS20351 and not in IS22330, in agreement with the decreased concentration of some carotenoids under severe drought stress [17, 38, 61].

The down-regulation of genes related to carotenoids and chlorophyll biosynthetic pathways leads to the down-regulation of light reaction and carbon fixation pathways, that in fact were dramatically affected by drought in the sensitive genotype IS20351. The decreased expression pattern mainly involved the light harvesting complex I and II and polypeptide subunits of the photosystems (I and II). In particular, the light-harvesting chlorophyll a/b-binding proteins (LHCBs) were extremely down-regulated in the sensitive genotype IS20351 according to several studies in which the down-regulation of LHCBs reduces plant tolerance [62–65]. The LHCBs, complexed with chlorophyll and xanthophylls, form the antenna complex [66] and play an important role in adaptation to environmental stress [63–65]. Their expression is regulated by multiple environmental factors including light [67], oxidative stress [68, 69] and abscisic acid (ABA) [70]. Also the genes involved in the “carbon fixation” were more greatly down-regulated in the sensitive genotype IS20351 rather than in the tolerant one. The up-regulation of Sb03g040610.1 was the main exception in the expression pattern of this genotype; this gene codes for the electron carrier ferrodoxin. Comparing the Log2 values of this gene in the two genotypes, it appears that this gene was more up-regulated in the sensitive genotype than in the tolerant one (5.2 and 3.4 for IS20351 and IS22330, respectively). This result indicates that the tolerant genotype IS22330 could better cope with the excess of light during drought stress. This is further supported at a physiological level by the low qNP value recorded. Conversely, the sensitive genotype IS20351 over expressed this gene so that it can dispose the excess of electrons and consequently waste the excess of light in non-photochemical reactions.

According to literature, under drought stress starch (inactive osmotically) content decreases, whilst content of soluble sugars (osmotically active) increases, assuring the maintenance of leaf water status and plant growth [23–25]. In the sensitive genotype IS20351, starch synthases were down-regulated and enzymes involved in the degradation of starch and sucrose up-regulated. According to Sturm and Tang [71] invertases play a role in several processes ranging from phloem loading to response to abiotic and biotic stresses [23, 72]. Exogenous ABA applied in soybean green beans [73] and maize leaves exposed to drought [74] showed an increase in invertase activity. Gazarrani and McCourt [75] also highlighted that hexose-based signals originating from sucrose cleavage are implicated in the regulation of ABA biosynthetic genes. It is well known that sucrose plays a crucial role as a key molecule in energy transduction and as a regulator of cellular metabolism [76–78]. Furthermore, sucrose and other sugars are energy and carbon sources required for defence response and are necessary for plant survival under drought stress conditions [79]. Like hormones, sucrose can act as primary messenger controlling the expression of several genes involved in sugar metabolism.

Lipids are important membrane components and, under drought stress, significant modifications of the lipid membranes occur. For this reason our investigation also focused on this metabolic pathway. The fatty acid elongation is considered to be the rate-limiting step in cuticular wax biosynthesis [80, 81]. The accumulation of wax has a key role in limiting water losses from plants [82]. It is widely accepted that drought stress can increase the amount of wax in several species [83–87] and that this increase is associated with an improved drought tolerance [88]. According to our results, the sensitive genotype IS20351 up-regulated these genes in response to drought; on the contrary, the drought tolerant genotype IS22330 remained unchanged. The hypothesis is that the tolerant genotype IS22330 has a constitutively higher expression level of genes related to drought tolerance, such as genes involved in cuticular wax synthesis and fatty acid desaturation. This hypothesis is also confirmed by the observation that, according to Torres-Martin et al. [89], no changes in omega-3 desaturase expression were highlighted in response to drought in the tolerant genotype IS22330. On the contrary, the omega-3 desaturases were down-regulated in the sensitive genotype IS20351 [89].

The first evidence of the involvement of sphingolipids in the signal-transduction pathways in plants, including in response to drought, was provided by Ng et al. [90]. Until that moment only the implication of sphingolipids in conferring stability to plant membranes, contributing to acclimation to drought stress had been hypothesized [91]. Spiegel and Milstien [92] afterwards explored the link between the sphingosine-1-phosphate and the drought hormone abscisic acid in the release of calcium from the vacuole. RNA-Seq results highlighted the ineffective response of the drought sensitive genotype IS20351 that down-regulated sphingolipids in response to drought, except for a ceramidase (sb03g028410.1).

In cowpea leaves a massive breakdown of membrane lipids was observed in response to drought with a more severe degradation in the sensitive plants [93]. The main enzyme responsible for the drought-induced degradation of membrane phospholipids is phospholipase D (PLD) [94]. According to El Masouf et al. [95], the drought sensitive genotype IS20351 strongly up-regulated the PLD expression, whilst in the drought tolerant IS22330 the expression was only slightly up-regulated. Recently, PLD up-regulation was associated to drought and salt stress tolerance [96–99] and the product of its activity, the phosphatidic acid, is involved in ABA signalling in stomatal movement [100]. PLDa1, in particular, is the most predominant PLD in plants activated by ABA [101].

Some interesting genes provided insight into the drought tolerance of the genotypes analysed. The Sb06g014320 gene, encoding for a glycerophosphodiester phosphodiesterase, found to be up-regulated in response to drought in sorghum leaves [12], was strongly down-regulated in response to drought in the sensitive genotype IS20351. The Sb07g027910 gene, encoding for a monogalactosyl-diacylglycerol (MGDG) synthase, found to map to a stay green QTL [102] and to be overexpressed in response to drought in sorghum leaves, was down regulated in the sensitive genotype IS20351. Since these genes are involved in drought tolerance related pathways, the first in choline biosynthesis and the second in phosphatidylinositol biosynthesis, a down regulation in response to drought is proof of sensitivity to drought stress for the sensitive genotype IS20351. A confirmation of the drought tolerance of IS22330 was the overexpression of genes related to the phosphatidylinositol biosynthesis, such as sb08g016610, sb08g022520 and sb05g026855.

## Conclusion

RNA-Seq analysis, performed in this study, proved to be a good method to investigate complex traits in different genotypes. The sorghum transcriptome analysed in response to drought conditions revealed unequivocal traits of sensitivity and tolerance in the two sorghum genotypes studied.

The first evidence of sensitivity to drought of the genotype IS20351 was represented by the physiological measurements (gas exchange and chlorophyll fluorescence) that drought dramatically affected. This evidence was confirmed at a transcriptomic level by the higher number of differentially expressed genes (DEGs) observed in the sensitive genotype IS20351 and not in the tolerant genotype IS22330. The sensitivity to drought of IS20351 was further confirmed by the lower constitutive expression level of “secondary metabolic process” (GO:0019748) and “glutathione transferase activity” (GO:000004364) observed under well-watered conditions in IS20351 in comparison with the tolerant genotype IS22330. In addition, the enriched GO terms analysis highlighted the differences existing between the two genotypes in coping with drought stress and the strategies adopted. The sensitive genotype hydrolysed carbohydrates and sugars, while the tolerant IS22330 activated the synthesis of other amino acids (glycinbetaine, glutathione) to cope with drought stress. In conclusion, we can confirm that the sensitive genotype IS20351 perceived the drought stress imposed (0.2 FTSW) to a greater extent than the tolerant genotype IS22330, showing an overactive genetic response. IS22330, on the other hand, being generally less affected by drought in all the analysed pathways, could be used as a genetic donor to further improve the sorghum germoplasm with drought tolerance traits.

## Methods

### Plant material, drought stress conditions and physiological measurements

Two sorghum genotypes of the *durra* race, IS20351 and IS22330, were cultivated in pots in July 2013 in a dry down experiment in open field condition in the experimental station of Università Cattolica del Sacro Cuore, Piacenza, Italy. The genotypes are part of germplasm collection of CIRAD and were provided by the CRB-T (Centre de Resources Biologiques Tropicales) CIRAD Montpellier. IS20351 and IS22330 were previously characterized in 2012 for their contrasting tolerance to drought [32]. According to Fracasso et al. [32], germination of seeds was carried out in Petri dishes at 25 °C and in dark conditions for 3 days. Five germinated seeds were planted in plastic pots (16 L capacity), filled with a base layer of sand to guarantee drainage and 8 kg of a soil mixture (24 % clay, 64 % silt, and 12 % sand), that had been previously sieved, dried and homogenized. At the 4th leaf stage, plants were thinned in order to have one healthy plant per pot.

The Fraction of Transpirable Soil Water (FTSW) was determined as the ratio of Available Soil Water Content (ASWC) divided by the Total Transpirable Soil Water (TTSW) as follows:

$$ FTSW=\frac{ASWC}{TTSW} = \frac{SWC-WP}{FC-WP} $$

Where ASWC represent the Available Soil Water Content for the plant, derived from the actual soil water content calculated as difference between the Soil Water Content (SWC) and the soil water content at Wilting Point (WP), and TTSW as the difference between the soil water content at Field Capacity (FC) and the water content at WP. Both FC and WP were determined in a short previous experiment (data not shown).

Plants were grown under well-water conditions until they reached the 6th leaf stage. At this moment, all the plants were irrigated until FC, the soil surface was covered by a thin layer of perlite, and the top of the pot was covered with PVC bags. A little slit was made in the bottom of the plastic bag to allow the sorghum plant to grow through. The slit was sealed with adhesive packing tape to minimise water loss by evaporation. Following the protocol of the dry-down experiment [32] a decrease of pot weight between two consecutive weight determinations is only attributed to plant transpiration.

Forty plants were divided in two groups: the well-watered (WW) and the drought stressed (DS) plants. Irrigation was withheld for half of them (the DS ones) till the FTSW value reached 0.2. This value was kept constant for nine days by re-integrating water losses of the DS plants day by day, while the WW plants were irrigated daily to maintain soil water content close to 0.7 FTSW. After nine days had passed, the plants were harvested in order to perform physiological and transcriptomic analysis.

Leaf area, chlorophyll fluorescence parameters (maximum quantum yield, Fv/Fm, photosystem II efficiency, ΦPSII, and non photochemical quenching, qNP), gas exchange measurements (photosynthetic rate, Pn, and transpiration, E) were measured for the entire duration of the experiment every two days. Pn and E data were used to calculate the intrinsic WUE (WUE_i_) as the ratio between photosynthetic activity and the transpiration rate (μmol mol^−1^). At the destructive sampling date (on the 42nd day after emergence, DAE), leaves samples were collected in order to perform transcriptomic analysis. At the same moment, relative water content (RWC) and biomass production were determined in order to calculate the agronomic water use efficiency (WUE_a_) the ratio between dry biomass production (g) and the total transpired water (L) according to Mastrorilli et al. [104].

Measurements of chlorophyll fluorescence parameters Fv/Fm, qNP and ΦPSII were carried out with a portable chlorophyll fluorometer (Fluorescence Monitoring System, Hansatech instruments, Norfolk, England) on the youngest fully expanded leaf. The value of minimal fluorescence was determined through pre-dawn measurements by applying weak modulated light (0.4 μmol m^−2^ s^−1^) and maximal fluorescence (Fm) was induced by a short pulse (0.7 s) of saturating light (15300 μmol m^−2^ s^−1^). The measurements were recorded between 12 and 2 pm for ΦPSII and before dawn for Fv/Fm.

Photosynthetic rate (Pn) was measured on the same leaf used for chlorophyll fluorescence measurements using a portable infrared gas analyser (CIRAS-2, PP System, Amesbury, USA): leaf surface area 4,5 cm^2^, saturated CO_2_ concentration of 400 μmolmol^−1^, and PPFD 2000 μmol m^−2^ s^−1^. Photosynthetic rate (Pn) was recorded between 12 and 2 pm.

Relative Water Content (RWC) was determined at the destructive sampling time according to the methodology described by Barr and Weatherley [103]. Twelve leaf disks of 20 mm of diameter were collected from each plant for the RWC determination. The disks were weighed, then soaked in distilled water for 24 h at 4 °C in the dark to determine the turgid weight. The dry weight was determined after drying the leaves for 72 h at 95 °C. The relative water content was then calculated using the following equation:

$$ RWC=\frac{\left(FM-DM\right)}{\left(TM-DM\right)}*100 $$

where FW is the fresh weight, TW the turgid weight after the rehydration in distilled water and DW the dry weight after drying.

### RNA extraction, cDNA library construction and sequencing

Three biological replicates were used for all RNA-Seq experiments from each genotypes and water treatment. The total RNA from the leaf meristem was extracted using Trizol reagent (Invitrogen, Carlsbad, CA) and purified using the RNeasy Plant Mini kit (Qiagen, Valencia, CA). On column DNase digestion was performed according to the manufacturer’s protocol (Qiagen, Valencia, CA). RNA quality and integrity was verified using a 2100 Bioanalyzer RNA Nanochip (Agilent, Santa Clara, CA) and all three samples had RNA Integrity Number (RIN) value more than 8.5. The quantification of the total RNA was checked by a NanoDropND-1000 Spectrophotometer (Nano-Drop, Wilmington, DE) and agarose gel electrophoresis.

Illumina sequencing using the GAII platform was performed at Beijing Genomics Institute (BGI-Shenzhen, Shenzhen, China http://www.genomics.cn/en/index) according to the manufacturer’s instructions (Illumina, San Diego, CA). Briefly, poly-A RNA was isolated from 20 μg of total RNA using Magnetic Oligo (dT) Beads (Illumina) and digested in short fragment. First and second strand synthesis were followed by end repair, and adenosines were added to the 3’ ends. Adapters were ligated to the cDNA and fragments (200 ± 25 bp) were purified by agarose gel electrophoresis and amplified by PCR. Finally, after validating on an Agilent Technologies 2100 Bioanalyzer using the Agilent DNA 1000 chip kit, the cDNA library was sequenced on a PE flow cell using Illumina Genome Analyzer IIx, and the workflow was as follows: template hybridization, isothermal amplification, linearization, blocking, sequencing primer hybridization, and sequencing on the sequencer for Read 1.

### Data processing and analysis

The RNA-seq reads generated by the Illumina Genome Analyzer were initially processed to remove the adapter sequences, reads in which unknown bases are more than 10 % and low-quality reads. After filtering, the remaining reads, so called “clean reads”, were used for downstream bioinformatics analysis. In the pipeline, clean reads are aligned to the reference sequence (ftp://ftp.ensemblgenomes.org/pub/plants/release-20/fasta/sorghum_bicolor/dna/) by using SOAPaligner/SOAP2. No more than 5 mismatches are allowed in the alignment. A quality control step was performed after that step and the distribution of reads on reference genes was analysed. Gene coverage was calculated as the percentage of a gene covered by reads. This value is equal to the ratio of the base number in a gene covered by unique mapping reads to the total base number of coding region in that gene. The expression level was, on the other hand, calculated using RPKM (Reads per Kilobase transcriptome per Million mapped reads) method [105], according to the following formula:

$$ RPKM = \frac{10^6C}{NL/{10}^3} $$

where C is the uniquely mapped counts determined from the high quality category, L is the cDNA length for the longest splice variant for a particular gene and N is the number of total mappable reads which was determined as the sum of the high quality reads and the highly repetitive reads. This method is able to eliminate the influence of different gene length and sequencing discrepancy on the calculation of gene expression. Log_2_ transformations of this normalization were performed.

### Screening, expression pattern, gene ontology analysis and pathway enrichment of DEGs

A strict algorithm was developed to identify differentially expressed genes between two samples and false positive and false negative errors are performed using Benjamini and Yekutieli [106] FDR method. We used FDR ≤0.001and the absolute value of Log_2_Ratio ≥2 as the threshold to judge the significance of gene expression difference. Gene Ontology (GO) enrichment was based on AgriGO software [107] with hypergeometric statistical test and Hocberg (FDR).

Pathway enrichment analysis of DEGs was performed using the Kyoto Encyclopedia of Genes and Genome (KEGG, http://www.genome.jp/kegg/). This analysis allows to identify enriched metabolic pathways or signal transduction pathways in DEGs comparing with the whole genome background. A strict algorithm was used for the analysis:

$$ P=1-{\displaystyle \sum_{i=0}^{m-1}}\frac{\left(\begin{array}{c}\hfill M\hfill \\ {}\hfill i\hfill \end{array}\right)\left(\begin{array}{c}\hfill N-M\ \hfill \\ {}\hfill n-i\hfill \end{array}\right)}{\left(\begin{array}{c}\hfill N\hfill \\ {}\hfill m\hfill \end{array}\right)} $$

Where N is the number of all genes with KEGG annotation; n is the number of DEGs in N, M is the number of all genes annotated to specific pathway. Pathways with Qvalue ≤0.05 are significantly enriched in DEGs.

### Novel transcript prediction and alternative splicing analysis

The assembled transcripts were compared with the annotated genomic transcripts from the reference sequences in order to discover novel transcribed regions. Three requirements are needed: the transcript must be at least 200 bp away from annotated gene, the length of the transcript must be over 180 bp, the sequencing depth must be no less than 2. The Coding Potential Calculator (CPC: http://cpc.cbi.pku.edu.cn/ ) was used to assess the protein-coding potential. TopHat software [108] was used to detect alternative splicing events (ASE).

### Ethics approval and consent to participate

Not applicable.

### Consent to publish

Not applicable.

### Availability of data and materials

The data sets supporting the results of this article are included within the article and its additional files.

### Availability of supporting data

The data discussed in this publication have been deposited in NCBI’s Gene Expression Omnibus (Edgar et al., 2002) and are accessible through GEO Series accession number GSE80699 (http://www.ncbi.nlm.nih.gov/geo/query/acc.cgi?acc=GSE80699).

## Additional files

Additional file 1: Table S1. Singular enrichment analysis using AgriGO to identify enriched gene ontologies among the 340 unique up-regulated genes in IS22330 under WW conditions. **Table S2.** Singular enrichment analysis using AgriGO to identify enriched gene ontologies among the 160 unique down-regulated genes in IS22330 under DS conditions. **Table S3.** Singular enrichment analysis using AgriGO to identify enriched gene ontologies among the 428 unique up-regulated genes resulted from the comparison of the two genotypes under DS conditions. **Table S4.** Singular enrichment analysis using AgriGO to identify enriched gene ontologies among the 393 unique down-regulated genes resulted from the comparison of the two genotypes under DS conditions. **Table S5.** Singular enrichment analysis using AgriGO to identify enriched gene ontologies among the 171 unique DEGs in response to DS conditions in both the genotypes. **Table S6.** Cross-comparison of enriched GO terms resulted from the unique up-regulated genes of IS20351 and IS22330 in response to DS conditions. **Table S7.** Cross-comparison of enriched GO terms resulted from the unique down-regulated genes of IS20351 and IS22330 in response to DS conditions. (XLSX 53 kb) Additional file 2: Table S1. Annotated genes belong to the most enriched metabolic pathways. (DOCX 57 kb)

## Abbreviations

ASE: alternative splicing events
DEGs: differentially expressed genes
DS: drought stressed
FC: field capacity
FTSW: fraction of transpirable soil water
Fv/Fm: maximum quantum yield of PSII
GO: gene ontology
KEGG: Kyoto Encyclopedia of Genes and Genomes
Pn: net photosynthetic rate
qNP: non-photochemical quenching
RPKM: reads per kilobase transcriptome per million mapped reads
RWC: Relative Water Content
SEA: single enrichment analysis
WP: wilting point
WUE: water use efficiency
WW: well-watered
ΦPSII: actual quantum yield of PSII.

## References

[1] Zhang HP, Kijne J, Barker R, Molden D (2003). Improving water productivity through deficit irrigation: examples from Syria, the North China Plain and Oregon, USA. Water productivity in agriculture: limits and opportunities for improvement.

[2] Rooney WL, Blumenthal J, Bean B, Mullet J (2007). Designing sorghum as a dedicated bioenergy feedstock. Biofuel, Bioprod Bioref.

[3] Dugas DV, Monaco MK, Olsen A, Klein RR, Kumari S, Ware D, et al. Functional annotation of the transcriptome of Sorghum bicolor in response to osmotic stress and abscisic acid. BMC Genomics. 2011;12:514–19. 10.1186/1471-2164-12-514 PMC3219791 22008187

[4] Paterson AH, Bowers JE, Bruggmann R, Dubchak I, Grimwood J, Gundlach H, et al. The Sorghum bicolor genome and the diversification of grasses. Nature. 2009;457(7229):551–56. 10.1038/nature07723 19189423

[5] Sanchez AC, Subudhi PK, Rosenow DT, Nguyen HT (2002). Mapping QTLs associated with drought resistance in sorghum (Sorghum bicolor L. Moench). Plant Mol Biol.

[6] Ashraf M (2010). Inducing drought tolerance in plants: recent advances. Biotechnol Adv.

[7] Mace ES, Rami JF, Bouchet S, Klein PE, Klein RR, Kilian A, et al. A consensus genetic map of sorghum that integrates multiple component maps and high-throughput Diversity Array Technology (DArT) markers. BMC Plant Biol. 2009;9:13–27. 10.1186/1471-2229-9-13 PMC2671505 19171067

[8] Rami JF, Dufour P, Trouche G, Fliedel G, Mestres C, Davrieux F, et al. Quantitative trait loci for grain quality, productivity, morphological and agronomical traits in sorghum (Sorghum bicolor L. Moench). Theor Appl Genet. 1998;97(4):605–16.

[9] Kebede H, Subudhi P, Rosenow D, Nguyen H (2001). Quantitative trait loci influencing drought tolerance in grain sorghum (*Sorghum bicolor* L. Moench). Theor Appl Genet.

[10] Buchanan CD, Lim S, Salzman RA, Kagiampakis I, Morishige DT, Weers BD, et al. Sorghum bicolor’s transcriptome response to dehydration, high salinity and ABA. Plant Mol Biol. 2005;58(5):699–720. 10.1007/s11103-005-7876-2 16158244

[11] Yazawa T, Kawahigashi H, Matsumoto T, Mizuno H (2013). Simultaneous transcriptome analysis of Sorghum and Bipolaris sorghicola by using RNA-seq in combination with de novo transcriptome assembly. PloSone.

[12] Pasini L, Bergonti M, Fracasso A, Marocco A, Amaducci S (2014). Microarray analysis of differentially expressed mRNAs and miRNAs in young leaves of sorghum under dry-down conditions. J Plant Physiol.

[13] Shakoor N, Nair R, Crasta O, Morris G, Feltus A, Kresovich S (2014). A Sorghum bicolor expression atlas reveals dynamic genotype-specific expression profiles for vegetative tissues of grain, sweet and bioenergy sorghums. BMC Plant Biol.

[14] Bekele WA, Wieckhorst S, Friedt W, Snowdon RJ (2013). High-throughput genomics in sorghum: from whole-genome resequencing to a SNP screening array. Plant Biotech J.

[15] Tuberosa R, Salvi S (2006). Genomics-based approaches to improve drought tolerance of crops. Trends Plant Sci.

[16] Chaves MM, Maroco JP, Pereira JS (2003). Understanding plant response to drought-from genes to the whole plant. Funct Plant Biol.

[17] Munne-Bosch S, Jubany-Mari T, Alegre L (2001). Drought-induced senescence is characterized by a loss of antioxidant defences in chloroplasts. Plant Cell Environ.

[18] Jubany-Mari T, Munne-Bosch S, Alegre L (2010). Redox regulation of water stress responses in field-grown plants. Role of hydrogen peroxide and ascorbate. Plant Physiol Biochem.

[19] Kyparissis A, Petropoulou Y, Manetas Y (2000). Summer survival of leaves in a soft-leaved shrub (Phlomis fruticosa L., Labiatae) under Mediterranean field conditions: avoidance of photoinhibitory damage through decreased chlorophyll contents. J Exp Bot.

[20] Kyparissis A, Drilias P, Manetas Y (2000). Seasonal fluctuations in photoprotective (xanthophyll cycle) and photoselective (chlorophylls) capacity in eight Mediterranean plant species belonging to two different growth forms. Funct Plant Biol.

[21] Havaux M, Tardy F (1999). Loss of chlorophyll with limited reduction of photosynthesis as an adaptive response of Syrian barley landraces to high-light and heat stress. Aust J Plant Physiol.

[22] Beck PA, Hutchison S, Gunter AS, Losi CT, Stewart BC, Capps KP, et al. Chemical composition and in situ dry matter and fibre disappearance of sorghum x Sudangrass hybrids. J Animal Sci. 2007;85:545–55. 10.2527/jas.2006-292 17235037

[23] Pelleschi S, Rocher JP, Prioul JL (1997). Effect of water restriction on carbohydrate metabolism and photosynthesis in mature maize leaves. Plant Cell Environ.

[24] Pinheiro C, Chaves MM, Ricardo CP (2001). Alterations in carbon and nitrogen metabolism induced by water deficit in the stems and leaves of Lupinus albus L. J Exp Bot.

[25] Yang J, Zhang J, Wang Z, Zhu Q, Wang W (2001). Remobilization of carbon reserves in response to water-deficit during grain filling of rice. Field Crops Res.

[26] Toumi I, Gargouri M, Nouairi I, Moschou P, Salem-Fnayou A, Mliki A, et al. Water stress induced changes in the leaf lipid composition of four grapevine genotypes with different drought tolerance. Biol Plant. 2008;52:161–4.

[27] Passioura J. Increasing crop productivity when water is scarce- from breeding to field management. In: ‘New directions for a diverse planet’. Proceedings of 4th International Crop Sciences Congress, Brisbane, Australia. 2004

[28] Cornic G (2000). Drought stress inhibits photosynthesis by decreasing stomatal aperture – not by affecting ATP synthesis. Trends Plant Sci.

[29] Chen TH, Murata N (2002). Enhancement of tolerance of abiotic stress by metabolic engineering of betaines and other compatible solutes. Curr Opin Plant Biol.

[30] Zhang J, Kirkham B (1996). Antioxidant responses to drought in sunflower and sorghum seedlings. New Phytol.

[31] Blum A. Drought resistance – is it really a complex trait? Func Plant Biol. 2011;38:753–57. 10.1071/FP11101 32480932

[32] Fracasso A, Trindade L, Amaducci S (2016). Drought tolerance strategies highlighted by two Sorghum bicolor races in a dry-down experiment. J Plant Phys.

[33] Usadel B, Poree F, Nagel A, Lohse M, Czedik-Eysenberg A, Stitt M (2009). A guide to using MapMan to visualize and compare Omics data in plants: a case study in the crop species, Maize. Plant Cell Env.

[34] Winkel-Shirley B (2002). Biosynthesis of flavonoids and effects of stress. Curr Opin Plant Biol.

[35] Ji W, Zhu L, Li Y, Yiang L, Zhao X, Cai H, et al. Overexpression of a glutathione S-transferase gene, GsGST, from wild soybean (*Glycine soja*) enhances drought and salt tolerance in transgenic tobacco. Biothecnol Lett. 2010;32:1173–79. 10.1007/s10529-010-0269-x 20383560

[36] Mano J, Inze D, Montago MV (2002). Early events in environmental stresses in plants induction mechanisms of oxidative stress. Oxidative stress in plants.

[37] Smirnoff N (1998). Plant resistance to environmental stresses. Curr Opin Plant Biol.

[38] Apel K, Hirt H (2004). Reactive oxygen species: metabolism, oxidative stress and signal transduction. Annu Rev Plant Biol.

[39] Schwanz P, Polle A (2001). Differential stress responses of antioxidative systems to drought in pedunculate oak (*Quercus robur*) and maritime pine (*Pinus pineaster*) grown under high CO2 concentrations. J Exp Bot.

[40] Ren J, Dai W, Xuan Z, Yao Y, Korpelainen H, Li C (2007). The effect of drought and enhanced UV-B radiation on the growth and physiological traits of two contrasting poplar species. Forest Ecol Manag.

[41] Liu C, Liu Y, Guo K, Fan D, Li G, Zheng Y, et al. Effect of drought on pigments, osmotic adjustment and antioxidant enzymes in six woody plant species in karst habitats of south western China. Environ Exp Bot. 2011;71:174–83.

[42] Agati G, Azzarello E, Pollastri S, Tattini M (2012). Flavonoids as antioxidants in plants: location and functional significance. Plant Sci.

[43] Ramakrishna A, Ravishankar GA (2011). Influence of abiotic stress signals on secondary metabolites in plants. Plant Signal Behavior.

[44] Grace S, Smirnoff N (2005). Phenolics as antioxidants. Antioxidants and reactive oxygen species in plants.

[45] Hernandez I, Alegre L, Munne-Bosch S (2006). Enhanced oxidation of flavan-3-ols and proanthocyanidin accumulation in water-stressed tea plants. Phytochemistry.

[46] Hernández I, Alegrel L, Munné-Bosch S (2004). Drought-induced changes in flavonoids and other low molecular weight antioxidants in *Cistus clusii* grown under Mediterranean field conditions. Tree Physiol.

[47] Tattini M, Galardi C, Pinelli P, Massai R, Remorini D, Agati G (2004). Differential accumulation of flavonoids and hydroxycinnmates in leaves of *Ligustrum vulgare* under excess light and drought stress. New Phytol.

[48] Castellarin SD, Pfeiffer A, Sivilotti P, Degan M, Peterlunger E, Di Gaspero G (2007). Transcriptional regulation of anthocyanin biosynthesis in ripening fruits of grapevine under seasonal water deficit. Plant Cell Environ.

[49] Agati G, Tattini M (2010). Multiple functional roles of flavonoids in photoprotection. New Phytol.

[50] Pollastri S, Tattini M (2011). Flavonols: old compounds for old roles. Ann Bot.

[51] Ma D, Sun D, Wang C, Li Y, Guo T (2014). Expression of flavonoid biosynthesis genes and accumulation of flavonoid in wheat leaves in response to drought stress. Plant Phys Biochem.

[52] Larson RA (1988). The antioxidants of higher plants. Phytochemistry.

[53] Ithal N, Reddy AR (2004). Rice flavonoid pathway genes, OsDfr and OsAns, are induced by hydration, high salt and ABA, and contain stress responsive promote elements that interact with the transcription activator, OsC1-MYB. Plant Sci.

[54] Hatier JHB, Gould KS (2008). Foliar anthocyanins as modulators of stress signals. J Theor Biol.

[55] Agati G, Stefano G, Biricolti S, Tattini M (2009). Mesophyll distribution of ‘antioxidant’ flavonoids glycosides in *Ligustrum vulgare* leaves under contrasting sunlight irradiance. Ann Bot.

[56] Agati G, Biricolti S, Guidi L, Ferrini F, Fini A, Tattini M (2011). The biosynthesis of flavonoids is enhanced similarly by UV radiation and root zone salinity in *L. vulgare* leaves. J Plant Physiol.

[57] Hernandez I, Alegre L, van Breusegem F, Munne-Bosch S (2009). How relevant are flavonoids as antioxidants in plants?. Trends Plant Sci.

[58] Tattini M, Remorini D, Pinelli P, Agati G, Saracini E, Traversi ML, Massai R (2006). Morpho-anatomical and biochemical adjustments in response to root zone salinity stress and high solar radiation in two Mediterranean evergreen shrubs, *Myrtus communis* and *Pistacia lentiscus*. New Phytol.

[59] Wolf L, Rizzini L, Stracke R, Ulm R, Rensing SA (2010). The molecular and physiological responses of *Physcomitrella patens* to ultraviolet-B radiation. Plant Physiol.

[60] Anjum F, Yaseen M, Rasool E, Wahid A, Anjum S (2003). Water stress in barley (*Hordeum vulgare* L.): on chemical composition and chlorophyll contents. Pak. J Agr Sci.

[61] Krammer I, Beckett RP, Wornik S, Zorn M, Pfeifhofer HW (2003). Revival of a resurrection plant correlates with its antioxidant status. Plant J.

[62] Andersson J, Walters RG, Horton P, Jansson S (2001). Antisense inhibition of the photosynthetic antenna proteins CP29 and CP26: implications for the mechanism of protective energy dissipation. Plant Cell.

[63] Andersson J, Wentworth M, Walters RG, Howard CA, Ruban AV, Horton P, Jansson S (2003). Absence of the Lhcb1 and Lhcb2 proteins of the light-harvesting complex of the photosystem II: effects on photosynthesis, grana stacking and fitness. Plant J.

[64] Ganeteg U, Kulheim C, Andersson J, Jansson S (2004). Is each light-harvesting complex protein important for plant fitness?. Plant Physiol.

[65] Kovacs L, Damkjær J, Kereiche S, Ilioaia C, Ruban AV, Boekema EJ, Jansson S, Horton P (2006). Lack of the lightharvesting complex CP24 affects the structure and function of the grana membranes of higher plant chloroplasts. Plant Cell.

[66] Jansson S (1994). The light-harvesting chlorophyll a/ b-binding proteins. Biochim Biophys Acta.

[67] Humbeck K, Krupinska K (2003). The abundance of minor chlorophyll a/b-binding proteins CP29 and LHCI of barley (*Hordeum vulgare* L.) during leaf senescence is controlled by light. J Exp Bot.

[68] Nott A, Jung HS, Koussevitzky S, Chory J (2006). Plastid-tonucleus retrograde signaling. Ann Rev Plant Biol.

[69] Staneloni RT, Rodriguez-Batiller MJ, Casal JJ (2008). Abscisic acid, high-light, and oxidative stress down-regulate a photosynthetic gene via a promoter motif not involved in phytochrome-mediated transcriptional regulation. Mol Plant.

[70] Weatherwax SC, Ong MS, Degenhardt J, Bray EA, Tobin EM (1996). The interaction of light and abscisic acid in the regulation of plant gene expression. Plant Physiol.

[71] Sturm A, Tang GQ (1999). The sucrose-cleaving enzymes of plants are crucial for development, growth and carbon partitioning. Trends Plant Sci.

[72] Zinselmier C, Westgate ME, Schussier JR, Jones R (1995). Low water potential disrupts carbohydrate metabolism in Maize (*Zea mays* L.) ovaries. Plant Physiol.

[73] Ackerson RC (1985). Osmoregulation in Cotton in response to water stress. III. Effects of phosphorus fertility. Plant Physiol.

[74] Trouverie J, Thévenot C, Rocher JP, Sotta B, Prioul JL (2003). The role of abscisic acid in response of a specific vacuolar invertase to water stress in the adult maize leaf. J Exp Bot.

[75] Gazarrani S, McCourt P (2001). Genetic interactions between ABA, ethylene and sugar signaling pathways. Curr Opin Plant Biol.

[76] Smeekens S, Rook F (1997). Sugar sensing and sugar-mediated signal transduction in plants. Plant Physiol.

[77] Smeekens S (2000). Sugar-induced signal transduction in plants. Ann Rev Plant Phys Plant Mol Biol..

[78] Martin SA, Facanha AR, Bressan-Smith RE. Developing Crop-Specific Irrigation Management Strategies Considering Effects of Drought on Carbon Metabolism in Plants, Water Quality, Soil and Managing Irrigation of Crops, Dr. Teang Shui Lee (Ed.), InTech, doi:10.5772/30419. Available from: http://www.intechopen.com/books/water-quality-soil-and-managing-irrigation-of-crops/developing-crop-specific-irrigation-management-strategies-considering-effects-of-drought-on-carbon-m.

[79] Bray EA (1997). Plant responses to water deficit. Trends Plant Sci.

[80] Millar AA, Kunst L (1997). Very-long-chain fatty acid biosynthesis is controlled through the expression and specificity of the condensing enzyme. Plant J.

[81] Denic V, Weissman JS (2007). A molecular caliper mechanism for determining very long-chain fatty acid length. Cell.

[82] Bartels D, Nelson D (1994). Approaches to improve stress tolerance using molecular genetic. Plant Cell Environ.

[83] Kosma KD, Bourdenx B, Bernard A, Parsons EP, Lü S, Joubès J, Jenks MA (2009). The impact of water deficiency on leaf cuticle lipids of Arabidopsis. Plant Physiol.

[84] Cameron KD, Teece MA, Smart LB (2006). Increased accumulation of cuticular wax and expression of lipid transfer protein in response to periodic drying events in leaves of tree tobacco. Plant Physiol.

[85] Samdur MY, Manivel P, Jain VK, Chikani BM, Gor HG, Desai S, Misra JB (2003). Genotypic differences and water-deficit induced enhancement in epicuticular wax load in peanut. Crop Sci.

[86] Jenks MA, Andersen L, Teusink RS, Williams MH (2001). Leaf cuticular waxes of potted rose cultivars as affected by plant development, drought and paclobutrazol treatments. Physiol Plant.

[87] Bondada BR, Oosterhuis DM, Murphy JB, Kim KS (1996). Effect of water stress on the epicuticular wax composition and ultrastructure of cotton (*Gossypium hirsutum* L.) leaf, bract, and boll. Environ Exp Bot.

[88] Islam MA, Du H, Ning J, Ye H, Xiong L (2009). Characterization of Glossy1- homologous genes in rice involved in leaf wax accumulation and drought resistance. Plant Mol Biol.

[89] Torres-Franklin ML, Repellin A, Huynh VB, d’Arcy-Lameta A, ZuilyFodil Y, Pham-Thi AT (2009). Omega-3 fatty acid desaturase (FAD3, FAD7, FAD8) gene expression and linolenic acid content in cowpea leaves submitted to drought and after rehydration. Environ Exp Bot.

[90] Ng CKY, Carr K, McAinsh MR, Powell B, Hetherington AM (2001). Drought-induced guard cell signal transduction involves sphingosine-1-phosphate. Nature.

[91] Norberg P, Mansson JE, Liljenberg C (1991). Characterisation of glucosylceramide from plasma membranes of plant root cells. Biochem Biophys Acta.

[92] Spiegel S, Milstein S (2000). Sphingosine-1-phosphate: signalling inside and out. FEBS Lett.

[93] Monteiro De Paula F, Pham Thi AT, Zuily-Fodil Y, Ferrari- Iliou R, Vieira Da Silva J, Mazliak P (1993). Effect of water stress on the biosynthesis and degradation of polyunsaturated lipid molecular species in leaves of *Vigna unguiculata*. Plant Physiol Biochem.

[94] Sahsah Y, Campos P, Gareil M, Zuily-Fodil FY, Pham-Thi AT (1998). Enzymatic degradation of polar lipids in Vigna unguiculata leaves and influence of drought stress. Physiol Plant.

[95] El Maarouf H, d’Arcy-Lameta A, Gareil M, Zuily-Fodil Y, Pham-Thi AT (2001). Cloning and expression under drought of cDNAs coding for two PI-PLCs in cowpea leaves. Plant Physiol Biochem.

[96] Bargmann BOR, Laxalt AM, ter Riet B, van Schooten B, Merquiol E, Testerink C, Haring M, Bartels D, Munnik T (2009). Multiple PLDs required for high salinity and water deficit tolerance in plants. Plant Cell Physiol.

[97] Hong Y, Pan X, Welti R, Wang X (2008). Phospholipase Da3 is involved in the hyperosmotic response in Arabidopsis. Plant Cell.

[98] Wang CR, Yang AF, Yue GD, Gao Q, Yin HY, Zhang JR (2008). Enhanced expression of phospholipase C1 (ZmPLC1) improves drought tolerance in transgenic maize. Planta.

[99] Wang X (2005). Regulatory functions of phospholipase D and phosphatidic acid in plant growth, development, and stress responses. Plant Physiol.

[100] Zhang M, Barg R, Yin M, Gueta-Dahan Y, Leikin-Frenkel A, Salts Y, Shabtai S, Ben-Hayyim G (2005). Modulated fatty acid desaturation via overexpression of two distinct omega-3 desaturases differentially alters tolerance to various abiotic stresses in transgenic tobacco cells and plants. Plant J.

[101] Hong Y, Zhang W, Wang X (2010). Phospholipase D and phosphatidic acid signalling in plant response to drought and salinity. Plant Cell Environ.

[102] Mace ES, Jordan DR (2011). Integrating sorghum whole genome sequence information with a compendium of sorghum QTL studies reveals uneven distribution of QTL and of gene-rich regions with significant implications for crop improvement. Theor App Gen.

[103] Barr HD, Weatherley PE (1962). A re-examination of the relative turgidity technique for estimating water deficit in leaves. Aust J Biol Sci.

[104] Mastrorilli M, Katerji N, Rana G (1995). Water efficiency and stress on grain sorghum at different reproductive stages. Agr Water Manag.

[105] Mortazavi A, Williams BA, McCue K, Schaeffer L, Wold B (2008). Mapping and quantifying mammalian transcriptomes by RNA-Seq. Nat Methods.

[106] Benjamini Y, Yekutieli D (2001). The control of false discovery rate in multiple testing dependency. Ann Stat.

[107] Du Z, Zhou X, Ling Y, Zhang Z, Su Z (2010). agriGO: a GO analysis toolkit for the agricultural community. Nucleic Acids Res.

[108] Trapnell C, Pachter L, Salzberg SL. TopHat: discovering splice junctions with RNA-Seq. Bioinformatics 2009; 25:1105-11. 10.1093/bioinformatics/btp120 PMC2672628 19289445

