# Supplementary material for: Drought stress tolerance strategies revealed by RNA-Seq in two sorghum genotypes with contrasting WUE
Source: BMC Plant Biol. 2016 May 21;16:115. doi: 10.1186/s12870-016-0800-x (PMC4875703; doi:10.1186/s12870-016-0800-x)
Supplement: Additional file 2: Table S1. — Annotated genes belong to the most enriched metabolic pathways. (DOCX 57 kb) [file 12870_2016_800_MOESM2_ESM.docx]

**Table S1: Annotated genes belong to the most enriched metabolic pathways.**

Annotation sources: (Zm) *Zea mays*, (At) *Arabidopsis thaliana*, and (Os) *Oryza sativa*. The extent of differential expression is measured as Log2 ratio. Values in blue and red indicate the fold decrease and increase in response to drought stress in the two genotype IS20351 and IS22330. Hyphen (-) indicates failure to meet significant cut-off or undetected.

|  |  |  |  |
| --- | --- | --- | --- |
| Gene ID | Annotation | IS20351 | IS22330 |
|  |  | Log2ratio | Log2ratio |
|  |  |  |  |
| **Antioxidant pathway** | |  |  |
| sb06g023440.1 | Thireodoxin family protein (Os) | 2.13 | - |
| sb01g036620.1 | Atypical Cys His rich thioredoxin 4 (Os) | 2.49 | - |
| sb04g033350.1 | Cytocrome B5 isoform E (heme binding) (Os) | 2.74 | - |
| sb09g001690.1 | Dehydroascorbate reductase 2 (At) | - | 2.14 |
| sb06g027520.2 | Ascorbate peroxidasel heme binding / peroxidase (Os) | -3.19 | - |
| sb06g001970.1 | L-ascorbate peroxidase (Os) | 3.79 | - |
| sb06g027520.1 | Ascorbate peroxidase,heme binding / peroxidase (Os) | -3.89 | - |
| sb01g042260.3 | Oxygen binding /transporter (Zm) | -4.64 | - |
| sb09g025730.2 | Oxygen binding /transporter (Os) | -6.74 | 2.28 |
| sb01g042260.2 | Oxygen binding /transporter (Zm) | -2.97 | - |
| sb08g017090.1 | Glutaredoxin family protein (At) | -4.36 | -3.23 |
| sb03g014790.1 | Electron carrierprotein disulfide oxidoreductase (At) | -3.62 | - |
| sb03g014800.1 | Glutaredoxin family protein (Os) | -2.80 | - |
| sb08g017170.1 | Glutaredoxin family protein (At) | -2.46 | - |
| sb08g017120.1 | Glutaredoxin family protein (Os) | -2.70 | - |
| sb02g040650.1 | Thioredoxin peroxidase (Os) | 4.94 | 4.06 |
| sb04g006270.1 | Protein peroxiredoxin-5 (Os) | -3.20 | - |
| sb10g002970.1 | Superoxide dismutase (Os) | -2.35 | - |
|  |  |  |  |
| **Secondary metabolism pathway** | |  |  |
| sb02g005410.1 | Protein 1-deoxy-D-xylulose-5-phosphate synthase (Os) | - | -4.04 |
| sb02g005380.1 | Protein 1-deoxy-D-xylulose-5-phosphate synthase (Os) | - | -6.52 |
| sb04g001810.1 | Protein terpene synthase 14 (At) | - | -2.47 |
| sb07g002990.1 | Protein terpene synthase 6 (Os) | - | -2.38 |
| sb04g001810.2 | Protein terpene synthase 14, S-linalool synthase (Os) | - | -3.20 |
| sb04g028050.1 | Geranylgeranyl reductase (Os) | -3.63 | - |
| sb02g037510.1 | Geranilgeranil pyrophosphate synthetase 1 GGPS1 (Os) | -2.10 | - |
| sb02g039970.1 | Protein oxidoreductase (Os) | -3.01 | - |
| sb10g031020.2 | Phytoene synthase, chloroplast precursor (Zm) | -2.35 | - |
| sb10g031020.1 | Phytoene synthase, chloroplast precursor (Zm) | -6.38 | - |
| sb03g026020.1 | Protein lycopene epsilon cyclase (Os) | -2.58 | - |
| sb05g022745.1 | Protein cycloartenol synthase CAS1(Os) | -8.41 | - |
| sb09g000980.1 | Terpene synthase 21 TPS21 (At) | -2.41 | - |
| sb09g001000.1 | Protein sesquiterpene cyclase (Os) | 2.24 | - |
| sb07g004485.1 | Protein terpene synthase 10 (Os) | -2.11 | - |
| sb07g005130.1 | Protein terpene synthase 6 (Os) | 3.90 | -3.21 |
| sb07g004470.1 | Protein terpene synthase 10 (Os) | -3.45 | -2.01 |
| sb05g026710.1 | Protein O-methyltransferase ZRP4 (Os) | - | 3.21 |
| sb10g005770.1 | Protein anthranilate N-benzoyltransferase protein 1 (Os) | - | -2.42 |
| sb10g005760.1 | Hydroxycinnamoyl CoA shikimate transferase HCT (At) | - | -2.48 |
| sb05g010100.1 | Protein O-methyltransferase ZRP4 (Zm) | - | 2.14 |
| sb02g024190.1 | Protein mannitol dehydrogenase (Os) | - | -4.58 |
| sb10g027340.1 | O-methyltransferase family 2 protein (At) | -4.95 | - |
| sb09g025530.1 | O-methyltransferase family 2 protein (Zm) | 2.98 | - |
| sb09g025510.1 | O-methyltransferase family 2 protein (At) | 4.10 | - |
| sb09g025540.1 | O-methyltransferase family 2 protein (At) | 2.06 | - |
| sb09g005480.1 | Protein 10-deacetylbaccatin III 10-O-acetyltransferase (Os) | 2.03 | - |
| sb10g000340.1 | Transferase family protein (At) | -4.24 | - |
| sb03g005860.1 | Protein O-methyltransferase ZRP4 (Zm) | -2.27 | - |
| sb10g027360.1 | Protein O-methyltransferase ZRP4 (Zm) | -4.44 | - |
| sb10g000330.1 | Protein transferase (At) | -3.33 | - |
| sb10g029620.1 | Protein O-methyltransferase ZRP4 (Os) | -2.22 | - |
| sb08g005125.1 | Protein O-methyltransferase ZRP4 (Os) | -2.76 | - |
| sb06g027990.1 | Protein transferase (Os) | 4.75 | - |
| sb02g006640.1 | Protein isoflavone-7-O-methytransferase 9 (Os) | -2.89 | - |
| sb06g001870.1 | Protein 10-deacetylbaccatin III 10-O-acetyltransferase (Os) | 3.79 | - |
| sb07g028520.1 | Caffeoyl-CoA 3-O-methyltransferase (At) | -3.98 | - |
| sb07g006090.1 | Cinnamyl alcohol dehydrogenase 9 CAD9 (At) | 2.27 | - |
| sb09g025140.1 | Protein tyrosine/DOPA decarboxylase 1 (Os) | - | -6.01 |
| sb07g003040.1 | Protein aromatic-L-amino-acid decarboxylase (Os) | -2.99 | - |
| sb01g022730.1 | Protein tyrosine decarboxylase 4 (OS) | 2.57 | - |
| sb04g026950.1 | Protein nitrilase 4 NIT4 (Os) | 2.41 | - |
| sb04g026940.2 | Protein nitrilase 4 NIT4 (Os) | 2.62 | - |
| sb04g026940.1 | Protein nitrilase 4 NIT4 (Os) | 2.03 | - |
| sb06g022320.1 | Protein eceriferum 1 CER1 (Os) | 2.04 | -2.91 |
| sb01g049060.1 | Membrane bound O-acyl transferase (MBOAT) family protein (At) | -2.38 | - |
| sb01g019590.1 | Protein octadecanal decarbonylase CER1 (Os) | 2.71 | 2.86 |
| sb04g026320.1 | Protein octadecanal decarbonylase CER1 (Os) | 3.32 | - |
| sb02g009850.1 | Chalcone and stilbene synthase (At) | 2.04 | - |
| sb10g005700.1 | Cinnamoyl-CoA reductase CCR1 (At) | 2.08 | - |
| sb03g028880.1 | Protein dihydroflavonol-4-reductase DFR (Os) | -2.99 | - |
| sb02g028590.1 | Protein dihydroflavonol-4-reductase DFR (Os) | 2.99 | - |
| sb10g027560.1 | Protein dihydroflavonol-4-reductase DFR (Os) | 2.70 | - |
| sb06g030140.1 | Transferase family protein (At) | - | -2.33 |
| sb05g020220.1 | Chalcone synthase 7 (Os) | - | -2.73 |
| sb10g005700.1 | Protein dihydroflavonol-4-reductase CCR1 (Os) | - | -2.50 |
| sb04g005510.1 | Cinnamoyl-CoA reductase CCR1 (At) | - | -2.20 |
| sb10g024520.1 | Cinnamoyl-CoA reductase family (At) | 4.87 | 8.98 |
| sb06g029520.1 | Protein leucoanthocyanidin reductase (Os) | 5.93 | 3.30 |
| sb03g008760.1 | Isoflavone reductase, putative (Zm) | -2.21 | -2.07 |
| sb04g027860.1 | Laccase 14 LAC14 (At) | 3.05 | - |
| sb09g022460.1 | Laccase LAC17 (At) | 2.35 | - |
| sb03g039520.1 | Laccase 17 LAC17 (At) | - | -2.43 |
| sb03g039570.1 | Laccase 2 LAC2 (Os) | - | -2.08 |
| sb03g039530.1 | Protein L-ascorbate oxidase precursor (Os) | - | -2.40 |
| sb10g022440.1 | L-ascorbate oxidase (Os) | 4.18 | 2.60 |
| sb03g039980.1 | Laccase LAC7 (At) | -8.09 | - |
| sb08g011530.1 | Protein copper ion binding protein (Os) | 4.05 | 3.48 |
|  |  |  |  |
| **Light reaction pathway** | |  |  |
| sb02g036260.1 | Chlorophyll A-B binding protein CP29 LHCB4 (At) | -2.17 | - |
| sb03g027030.1 | Protein chlorophyll a-b binding protein 2 (Os) | -3.63 | - |
| sb02g037410.2 | Chlorophyll a-b binding protein (At) | -2.41 | - |
| sb03g027040.1 | Protein chlorophyll a-b binding protein 2 (Os) | -3.20 | - |
| sb06g032690.1 | Light harvesting complex PSII subunit 6 (At) | -2.87 | - |
| sb05g007070.1 | Light harvesting complex PSII subunit 5 (At) | -2.95 | - |
| sb01g049040.1 | PsbP-like protein 1 (At) | -2.61 | - |
| sb02g035610.1 | Ferredoxin-NADP reductase-binding protein (Zm) | -3.46 | - |
| sb04g026720.1 | Photosystem II calcium ion binding protein (Os) | 2.47 | 2.11 |
| sb01g036240.1 | Photosystem II 11 kD protein (Os) | -2.91 | - |
| sb02g002830.1 | PSBR -photosystem II subunit R (At) | 4.47 | 3.65 |
| sb06g022890.1 | Photosystem II calcium ion binding protein (Os) | 2.61 | - |
| sb07g021260.3 | Light harvesting complex PSI subunit A4 (At) | -2.43 | - |
| sb02g037410.3 | Chlorophyll binding protein LHCA2 (At) | -3.29 | - |
| sb02g032815.1 | Photosystem I subunit P PSI-P(At) | -2.65 | - |
| sb03g035235.1 | Protein thylakoid membrane phosphoprotein 14 kda (Os) | -2.03 | - |
| sb06g016090.1 | Photosystem I subunit O PSI-O (At) | -2.23 | - |
| sb02g034570.2 | ATP synthase subunit gamma (Zm) | -2.13 | - |
| sb03g040610.1 | Ferredoxin-6 (Zm) | 5.22 | 3.37 |
|  |  |  |  |
| **Calvin cylce pathway** | |  |  |
| sb01g037510.1 | Protein CP12-2 (Os) | 3.17 | - |
| sb05g003480.1 | RuBisCO small chain (Os) | -2.22 | - |
| sb06g018880.6 | Glyceraldehyde-3-phosphate dehydrogenase (Zm) | -3.01 | - |
| sb06g018880.3 | Glyceraldehyde-3-phosphate dehydrogenase (Zm) | -2.83 | - |
| sb05g004590.1 | Fructose-bisphosphate aldolase, putative (Os) | -3.00 | - |
| sb01g039980.1 | D-fructose-1,6-bisphosphate 1-phosphohydrolase (Os) | -2.33 | - |
| sb06g004280.1 | Transketolase, putative (Os) | 2.82 | - |
| sb01g021920.1 | Protein chitinase 2, putative, (Os) | -2.89 | - |
| sb05g027880.1 | ADP/ ATP binding enzyme, RuBisCO activator (Os) | 5.98 | - |
|  |  |  |  |
| **Lipid pathway** |  |  |  |
| sb02g009540.1 | Fatty acyl-ACP thioesterases B FATB (At) | 3.92 | - |
| sb10g023190.1 | Fatty acyl-ACP thioesterases B FATB (At) | 2.14 | - |
| sb10g009610.1 | Acyl activating enzyme 5 AAE5 (At) | 2.73 | - |
| sb10g012080.1 | Acyl activating enzyme 1 AAE1 (At) | 3.16 | - |
| sb05g021840.1 | Long chain acyl CoA synthetase 2 LACS2 (At) | - | 3.04 |
| sb05g025040.1 | 3 Ketoacyl CoA synthetase 2 KCS2 (fatty acid elongase) (At) | -2.56 | -2.69 |
| sb09g029260.1 | 3 Ketoacyl CoA synthetase 11 KCS11 (At) | 3.12 | 3.58 |
| sb06g012520.1 | Acyl-acyl-carrier-protein desaturase (Os) | 2.01 | - |
| sb08g022360.1 | Fatty acid hydroxylase 1 FAH1 (Os) | - | -2.02 |
| sb04g029900.1 | Fatty acid desaturase 2 (omega-6 fatty acid desaturase) FAD2 (Os) | -3.3 | - |
| sb08g000460.1 | Fatty acid desaturase 8 (omega-3 fatty acid desaturase) FAD8 (Os) | 3.24 | - |
| sb04g029920.2 | Fatty acid desaturase 2 (omega-6 fatty acid desaturase) FAD2 (At) | -2.62 | - |
| sb04g029920.1 | Fatty acid desaturase 2 (omega-6 fatty acid desaturase) FAD2 (Os) | -2.38 | - |
| sb07g021640.1 | Fatty acid desaturase 6 (omega-6 fatty acid desaturase) FAD6 (At) | -2.07 | - |
| sb03g031940.1 | Phosphoethanolamine N-methyltransferase XPL1 (At) | -2.2 | - |
| sb01g042150.1 | UbiE/COQ5 methyltransferase family protein (Sb) | -2.7 | - |
| sb08g016610.1 | Phospholipid synthesis protein 1 PLS1 (Zm) | - | -2.23 |
| sb01g032250.1 | Diacylglycerol kinase 1 DGK1 (At) | 2.16 | - |
| sb02g038840.1 | Sulfotransferase family protein (At) | -3.73 | - |
| sb01g011900.1 | Desulfoglucosinolate sulfotransferase (At) | 2.13 | - |
| sb04g031660.1 | Hydroxyjasmonate sulfotransferase (At) | 4.13 | - |
| sb05g026855.1 | Flavonol sulfotransferase-like protein (Os) | - | 4.27 |
| sb04g029190.1 | ASC1-like protein 2 (Os) | -2.02 | - |
| sb03g036460.1 | Diacylglycerol kinase DGK (Os) | -3.11 | - |
| sb04g000940.1 | Sphingoid base hydroxylase 2 SBH2 (At) | -9.24 | - |
| sb04g000890.1 | Sphingoid base hydroxylase 2 SBH2 (At) | -4.13 | - |
| sb03g028410.1 | Ceramidase family protein (At) | 2.97 | - |
| sb01g032960.1 | 3-beta-hydroxy-delta5-steroid dehydrogenase (At) | -2.05 | -9.5 |
| sb01g032970.1 | 3-beta-hydroxy-delta5-steroid dehydrogenase (At) | - | -2.27 |
| sb03g006300.1 | Lecithin: cholesterol acyltransferase family protein (At) | 2.87 | - |
| sb03g006290.1 | Lecithin: cholesterol acyltransferase family protein (At) | 3.08 | - |
| sb05g025990.1 | Lipase class 3 family protein (At) | -3.11 | - |
| sb03g003410.1 | Lipase class 3 family protein (At) | 3.29 | - |
| sb01g028880.1 | Triacylglycerol lipase like protein (Os) | - | -2.73 |
| sb09g001420.1 | Lipase class 3 family protein (At) | - | -3.2 |
| sb08g022520.1 | Phospholipase D alpha 1 PLD Alpha 1 (Zm) | 4.44 | 2.46 |
| sb02g024910.1 | Phospholipase D alpha 2 PLD Alpha 2 (At) | - | -2.4 |
| sb09g030200.1 | Acyl-protein thioesterase 2 (Os) | -2.9 | - |
| sb06g019730.1 | Glycerophosphoryl diester phosphodiesterase family protein (Os) | -2.04 | - |
| sb07g026000.1 | Glycerophosphodiester phosphodiesterase (At) | -3.74 | - |
| sb01g015000.1 | Glycerophosphoryl diester phosphodiesterase precursor (Os) | -2.4 | - |
| sb06g014320.1 | Glycerophosphodiester phosphodiesterase ( At) | -2.38 | - |
| sb05g023970.2 | Acyl-CoA oxidase 2 ACX2 (At) | 2.52 | - |
| sb05g023970.1 | Acyl-CoA oxidase 2 ACX2 (At) | 2.95 | - |
| sb04g037280.2 | Peroxisomal 3-ketoacyl-CoA thiolase 3 PKT3 (At) | 2.22 | - |
| sb05g005330.1 | Fatty acid reductase 4 FAR4 (At) | 2.44 | - |
| sb07g024290.1 | Fatty acyl CoA reductase 1 FAR1 (At) | -2.96 | - |
| sb07g027910.1 | 1,2-diacylglycerol 3-beta-galactosyltransferase MGD2 (At) | -5.92 | - |
| sb03g006480.1 | Sulfoquinovosyldiacylglycerol 2; UDP-glycosyltransferase SQD2 (At) | -4.56 | - |
|  |  |  |  |
| **Carbon metabolism pathway** | |  |  |
| sb02g009870.1 | Granule -bound starch synthase 1b (Os) | -2.12 | - |
| sb04g021540.1 | 1,4-alpha-glucan branching enzyme IIB (Zm) | 2.35 | - |
| sb07g001320.1 | Fructokinase-2 (Zm) | -8.79 | - |
| sb03g013420.1 | Protein beta-fructofuranosidase (Os) | - | -4.15 |
| sb04g021810.2 | Beta-fructofuranosidase, cell wall Invertase 2 (Os) | -8.47 | - |
| sb03g047060.1 | Beta-fructofuranosidase, cell wall Invertase 4 (Os) | -2.48 | - |
| sb06g031930.1 | Beta-fructofuranosidase, cell wall Invertase 7 (Os) | 2.35 | - |
| sb06g031930.1 | Beta-fructofuranosidase, vacuolarl Invertase 7 (Os) | 2.35 | - |
| sb09g005840.1 | Hexokinase 1 HXK1 (Os) | 2.32 | - |
| sb01g035890.1 | Sucrose synthase 2 SUS2 (Os) | 2.28 | - |
| sb03g032830.1 | Alpha-amylase isozime 2 precursor AMY3 (Os) | 2.17 | -2.61 |
| sb02g035600.1 | Beta amylase BAM (Zm) | -2.84 | - |
| sb01g019850.1 | Beta amylase BAM (Os) | 2.80 | - |
